# Dual‐Dynamically Crosslinked Nanogels Enable Prolonged Ocular Retention for Multimodal Dry Eye Therapy

**DOI:** 10.1002/advs.78237

**Published:** 2026-10-11

**Authors:** Yanru Xin, Sisi Chen, Shengli Huang, Mengqi Huang, Jingyan Zhu, Zijian Wang, Jianliang Shen, Qinxiang Zheng

**Affiliations:** ^1^ Department of Ophthalmology Sir Run Run Shaw Hospital Zhejiang University School of Medicine Hangzhou People's Republic of China; ^2^ Zhejiang Key Laboratory of Ophthalmic Drug Discovery and Medical Device Research Eye Hospital Wenzhou Medical University Wenzhou People's Republic of China; ^3^ Zhejiang Engineering Research Center for Tissue Repair Materials Wenzhou Institute University of Chinese Academy of Sciences Wenzhou People's Republic of China; ^4^ National Clinical Research Center for Ocular Diseases Eye Hospital Wenzhou Medical University Wenzhou People's Republic of China

**Keywords:** anti‐inflammation, anti‐oxidation, dry eye disease, dual‐dynamically crosslinked, nanogels, precorneal retention

## Abstract

Dry eye disease (DED), a chronic disorder affecting nearly half the global population, is perpetuated by oxidative stress, inflammation, and tear film instability. Constrained by short precorneal residence and monotherapy, DED therapies necessitate frequent dosing and polypharmacy, leaving unmet needs for prolonged ocular retention and multimodal intervention. Herein, we develop berberine (BBR)‐loaded dual‐dynamically crosslinked nanogels (BBR@MPN‐NGs) integrating tannic acid (TA)‐zinc metal‐polyphenol networks with carboxymethyl chitosan/aldehyde‐functionalized poly(ethylene glycol) Schiff base networks, enabling a single‐platform multimodal therapy combining antioxidant, anti‐inflammatory, and lubricating capacities. Capitalizing on dual‐dynamic synergy, BBR@MPN‐NGs exhibit 1‐year colloidal and antioxidant stability alongside lubricity, while leveraging TA‐mediated adhesion and Schiff base reversibility to sustain corneal BBR exposure for 24 h, enabling once‐daily dosing. In murine DED models, BBR@MPN‐NGs outperform first‐line cyclosporine A and dexamethasone in promoting corneal epithelial repair and tear secretion. Mechanistically, the nanogels reprogram the pathological microenvironment by scavenging reactive oxygen species, attenuating pro‑inflammatory macrophage polarization, and suppressing c‐Jun N‐terminal kinase signaling to reduce pro‐inflammatory gene expression. Notably, the preservative‐free nanogels passed sterility and endotoxin testing, showing no ocular or systemic toxicity, which supports biosafety. These dual‐dynamically crosslinked nanogels enable reduced‐frequency and shortened treatment duration, offering an integrated strategy for complex ocular surface diseases requiring sustained multimodal intervention.

## Graphical abstract

This berberine‐loaded dual‐dynamically crosslinked nanogel integrates tannic acid‐zinc metal‐phenolic networks and carboxymethyl chitosan/aldehyde‐functionalized poly(ethylene glycol) dynamic Schiff base networks, achieving 1‐year colloidal and antioxidant stability, sustained corneal berberine exposure for 24 h to support convenient once‐daily dosing, and shortened treatment duration, thereby disrupting the vicious cycle of dry eye disease via multimodal therapy that combines antioxidant, anti‐inflammatory, and lubricating capacities. (Created with Adobe Illustrator).

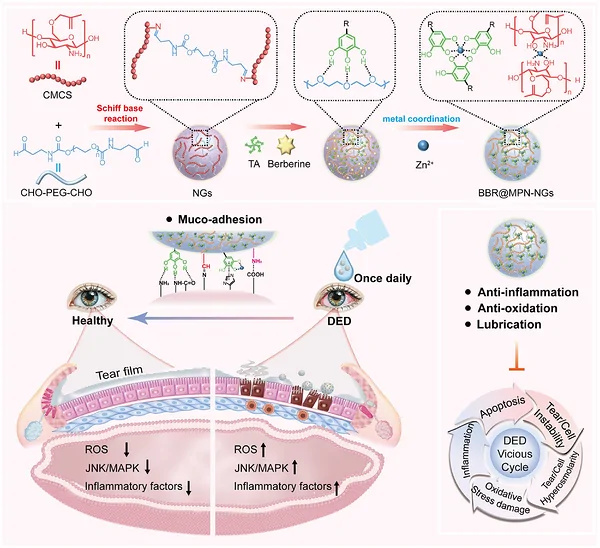

## 1 Introduction

Dry Eye Disease (DED), a multifactorial ocular surface disorder affecting millions globally, has escalated into a pressing public health concern, driven by the ubiquitous use of digital devices and environmental pollution [1, 2]. The core pathogenesis of DED involves the vicious cycle formed by oxidative stress, inflammatory responses, and tear film instability [3]. Current clinical management remains suboptimal, predominantly relying on artificial tears, immunosuppressive agents, and anti‐inflammatory agents, which target only isolated nodes within the pathological cascade. Artificial tears offer mere physical lubrication devoid of bioactivity [4], while immunosuppressive agents like cyclosporine A (CsA) suffer from a notoriously delayed onset, often requiring 3–6 months of continuous administration [5, 6]. Although corticosteroids such as dexamethasone (DEX) provide rapid symptomatic relief, their long‐term use is severely compromised by sight‐threatening complications, including glaucoma and cataracts [7]. Critically, most formulations rely on toxic preservatives such as benzalkonium chloride (BAC) that exacerbate ocular surface damage and goblet cell dysfunction [8]. Consequently, there is an urgent unmet need for the development of safe and multimodal therapeutic strategies for dry eye disease.

Topical eye drops remain the preferred clinical modality for managing DED due to their non‐invasive administration and minimal systemic exposure [9]. However, physiological barriers, including blinking, tear turnover, and nasolacrimal drainage, together with the mucus barriers rich in mucin, lipid, and protein, severely hinder precorneal residence, compromising bioavailability [10, 11]. Sustaining therapeutic efficacy necessitates patients adhering to burdensome regimens of frequent daily instillations and complex polypharmacy [12, 13]. In response to this clinical challenge, advanced delivery platforms have been engineered to overcome the ocular mucus barrier [14, 15, 16, 17, 18, 19, 20, 21]. Among them, polysaccharide‐based nanogels have attracted particular interest as ocular delivery systems owing to their high‐water retention, biocompatibility, and mucoadhesiveness [22]. Strategies such as chitosan‐modified liposomes leverage electrostatic interactions to enhance mucoadhesion [16], while dynamic Schiff base networks form covalent bonds with free amino groups on mucosal surfaces to improve ocular residence [17]. More recently, an ocular‐adhesive thermo‐responsive FK506 hydrogel [23] and porous microparticle eye drops carrying mesenchymal stem cells [24] have demonstrated prolonged ocular retention and improved therapeutic outcomes in murine DED models. Rational structural engineering of nanocarriers, including the design of hollow and porous nanoarchitectures, has further expanded the capacity for drug loading and controlled release [25]. Despite these advances, frequent instillation remains necessary for most eye drop formulations, and few platforms simultaneously address ocular retention and active modulation of the pathological microenvironment.

Metal‐phenolic networks (MPNs), inspired by mussel foot proteins and combining mucosal adhesion with intrinsic bioactivity, have emerged as a promising alternative to these biologically inert carriers [26, 27, 28, 29, 30]. Through hydrogen bonding with glycoproteins, MPNs readily adhere to mucosal surfaces, and their ability to scavenge reactive oxygen species (ROS) alleviates oxidative stress, directly addressing the pathological hallmarks of DED [31, 32]. In the ocular context, MPN‐based systems have so far been formulated mainly as coatings or bulk depots [13, 33], and coordination‐crosslinked nanogels explored for dry eye therapy still rely on a single network [34], leaving multifunctional integration within structurally defined nanoparticles insufficiently addressed. Moreover, conventional MPNs suffer from excessively rapid coordination kinetics, which hinder precise nanoscale morphological control [30, 35, 36, 37, 38]. They are also prone to structural dissociation under physiological conditions, thereby compromising long‐term stability and sustained therapeutic efficacy [39]. To overcome these limitations, dually crosslinked strategies offer a robust approach. Through a sacrificial bond mechanism, these systems form synergistic interlocking networks that impart superior structural stability and resistance to environmental disturbances [40, 41, 42, 43]. Given that dually crosslinked strategies effectively reinforce carrier structural stability and precisely modulate release kinetics, we hypothesized that integrating dynamic Schiff base networks with MPNs at the nanoscale would yield stable, mucoadhesive biomimetic nanogels suitable as eye drops for sustained intervention in the pathological microenvironment. To implement this concept, we introduced aldehyde‐functionalized poly(ethylene glycol) (PEG‐CHO) as a bifunctional regulator. PEG‐CHO participates in constructing dynamic covalent networks, while its steric hindrance prevents interparticle crosslinking. This dual function suppresses heterogeneous aggregation and macroscopic gelation, enabling the controlled synthesis of structurally uniform and stable dually crosslinked nanogels [44, 45]. For therapeutic integration, berberine (BBR), a clinically established drug for intestinal infections with a high safety profile, has been demonstrated to profoundly suppress DED‐associated inflammatory cascades via modulation of the PI3K/AKT/NF‐κB and mitogen‐activated protein kinase (MAPK) signaling pathways [46], thereby complementing the nanogel's antioxidant functionality with targeted anti‐inflammatory action.

Herein, we report the rational design of BBR‐loaded dual‐dynamically crosslinked nanogels (BBR@MPN‐NGs) that integrate metal‐phenolic coordination with dynamic Schiff base chemistry, successfully translating this design principle into a functional platform for prolonged ocular retention and multimodal DED therapy (Figure 1). In this framework, FDA‐approved tannic acid (TA) coordinates with the essential trace element zinc ions (Zn^2+^) to construct MPN networks with potent antioxidant capacity. Concurrently, carboxymethyl chitosan (CMCS), which possesses water solubility, excellent biocompatibility, and lubricating properties [47], reacts with PEG‐CHO to establish a dynamic Schiff base network. Liquid chromatography‐tandem mass spectrometry (LC‐MS/MS) analysis confirmed sustained corneal BBR exposure for up to 24 h, supporting the once‐daily dosing regimen. Building on this sustained corneal drug exposure, the platform enables multimodal therapy that integrates antioxidant, anti‐inflammatory, and lubricating capacities within a single nanogel platform, coupled with BBR release to support prolonged corneal surface therapy. Systematic characterization confirms exceptional lubricity and colloidal stability for up to 1 year, with TA content remaining above 90% of its initial value and BBR retention exceeding 90% after 12 months of storage. In vitro, the nanogels effectively mitigate oxidative stress and attenuate inflammatory factor expression by regulating the c‐Jun N‐terminal kinase/MAPK (JNK/MAPK) pathway, thereby preventing corneal epithelial cell death. In murine DED models, BBR@MPN‐NGs deliver multimodal therapeutic effects by promoting corneal repair, increasing goblet cell density, and restoring tear secretion and tear film stability. Notably, once‐daily administration achieves therapeutic efficacy comparable to that of frequent dosing regimens, outperforming clinical standards such as CsA and DEX, substantially improving patient compliance in chronic DED management. Furthermore, the preservative‐free nanogels pass sterility and endotoxin testing, showing no ocular or systemic toxicity, which further supports biosafety. These findings suggest that the unified dual‐dynamically crosslinked nanogel eye drops, by enabling less frequent dosing and a shorter treatment duration, represent an integrated strategy for sustained multitarget management of complex ocular surface diseases such as DED.

**FIGURE 1:**
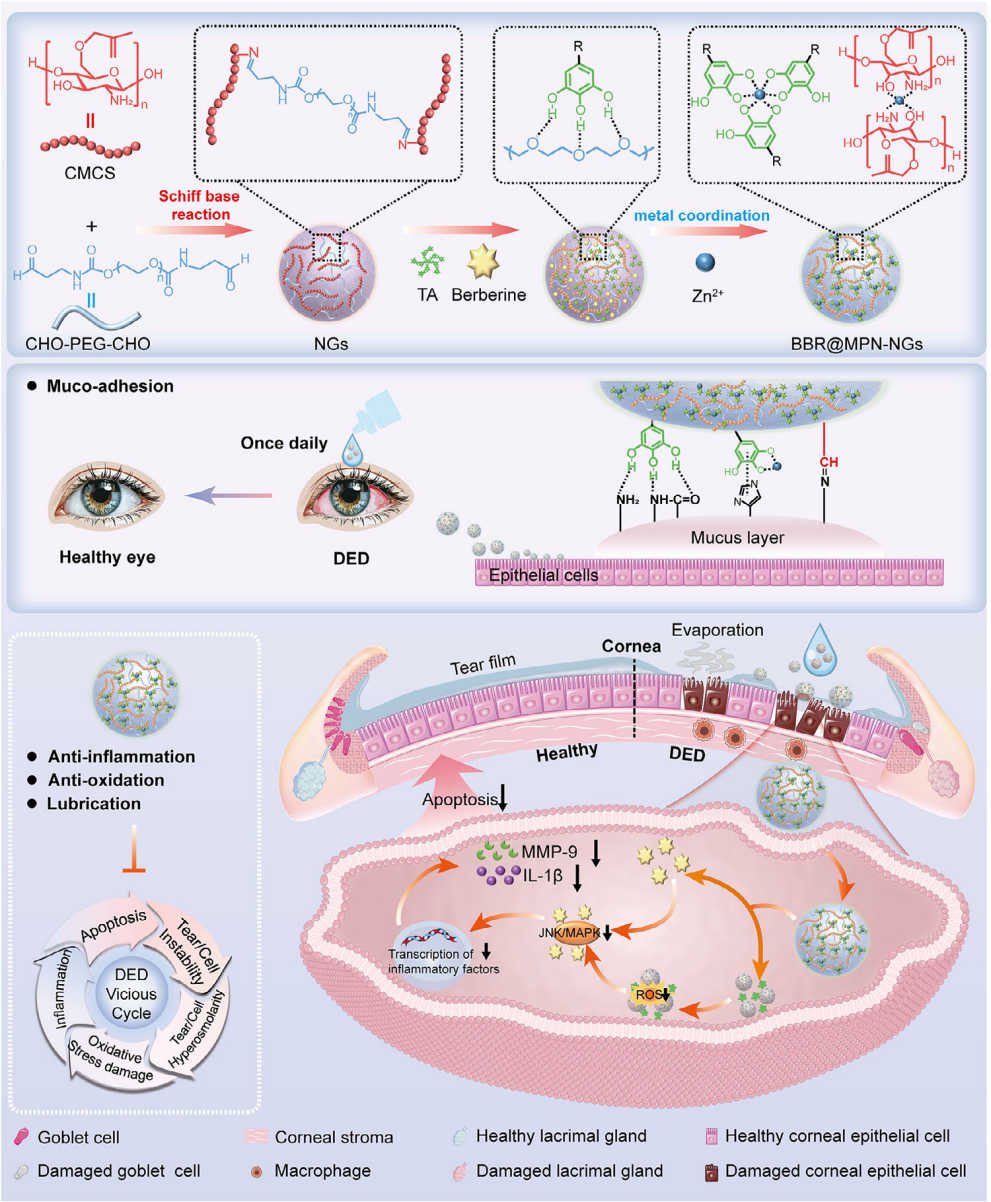
Schematic illustration of the preparation of synergistic dual‐dynamically crosslinked nanogels for prolonged ocular retention and multimodal dry eye therapy via combined anti‐inflammatory, antioxidant, and lubricating capacities. Created with Adobe Illustrator.

## 2 Results and Discussion

### 2.1 Fabrication and Characterization of the BBR@MPN‐NGs

BBR@MPN‐NGs were synthesized via a stepwise coordination and crosslinking process (Figure 1). CMCS and PEG‐CHO were initially dispersed in deionized water to form the nanogel backbone via Schiff base formation. TA, BBR, and Zn^2+^ were subsequently introduced, followed by pH adjustment to 7.0 to facilitate stable dual‐dynamically crosslinked networks. Dynamic light scattering (DLS) was employed to monitor particle size while varying the BBR feed ratio to optimize the formulation (Figure 2C, E). The blank nanogels MPN‐NGs (without BBR) exhibited a hydrodynamic diameter of approximately 200 nm. At a TA:BBR molar ratio of 1:0.25, the nanogels displayed the smallest size (∼220 nm) among all BBR‐loaded formulations, a polydispersity index (PDI) below 0.1, and a zeta potential of −22.24 mV (Figure S1). This ratio was selected for all subsequent experiments to balance the antioxidant activity of TA and the anti‐inflammatory efficacy of BBR while maintaining excellent colloidal dispersibility. Transmission electron microscopy (TEM) revealed a uniform spherical morphology (Figure 2A), and TEM‐energy‐dispersive x‐ray spectroscopy (TEM‐EDX) elemental mapping confirmed the homogeneous distribution of C, O, Zn, and N throughout the nanogel matrix (Figure 2B). Ultraviolet‐visible (UV/vis) absorption spectroscopy verified successful BBR encapsulation, as the characteristic absorption peak of BBR was preserved in the BBR@MPN‐NGs spectrum (Figure 2D).

**FIGURE 2:**
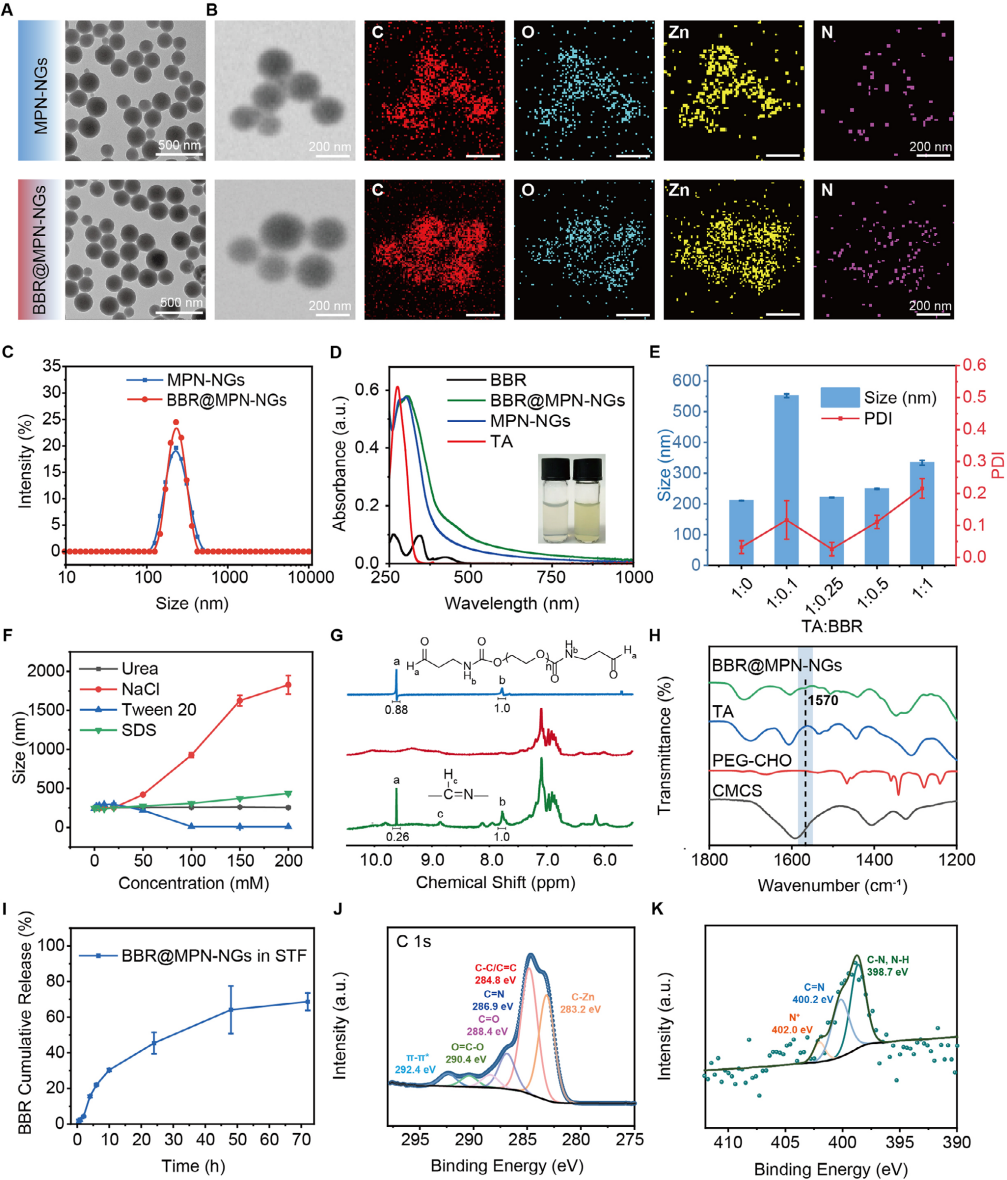
Characterization of BBR@MPN‐NGs. (A) Representative TEM images of MPN‐NGs and BBR@MPN‐NGs. Scale bar: 500 nm. (B) EDX elemental mapping of C, O, Zn, and N for MPN‐NGs and BBR@MPN‐NGs. Scale bar: 200 nm. (C) Hydrodynamic diameter distributions of MPN‐NGs and BBR@MPN‐NGs. (D) UV/vis absorption spectra of BBR@MPN‐NGs, MPN‐NGs, free TA, and free BBR. (E) Particle size of BBR@MPN‐NGs prepared at different TA:BBR molar ratios. (F) Stability assay of BBR@MPN‐NGs in the presence of NaCl, Urea, Tween 20, or SDS. Data are mean ± standard deviation (SD, *n* = 3). (G) ^1^H NMR spectra of PEG‐CHO (blue curve), BBR@MPN(OCH_3_) (red curve) and BBR@MPN‐NGs (green curve). (H) FTIR spectra of BBR@MPN‐NGs, TA, PEG‐CHO, and CMCS in the region of 1200–1800 cm^−1^. (I) In vitro drug release profile of BBR@MPN‐NGs in simulated tear fluid containing 1% SDS. Data are the mean ± SD (*n* = 3). (J) High‐resolution XPS spectrum of BBR@MPN‐NGs in the C 1s region. (K) High‐resolution XPS spectrum of BBR@MPN‐NGs in the N 1s region.

The assembly mechanism of BBR@MPN‐NGs was systematically investigated using DLS, attenuated total reflection Fourier transform infrared spectroscopy (ATR‐FTIR), proton nuclear magnetic resonance (^1^H NMR), and X‐ray photoelectron spectroscopy (XPS). To identify the dominant non‐covalent interactions, the colloidal stability of the nanogels was evaluated in the presence of sodium dodecyl sulfate (SDS), Tween 20, NaCl, and urea. Figure 2F showed that pronounced size changes upon exposure to NaCl and surfactants (SDS/Tween 20), but not urea, indicated that electrostatic and hydrophobic forces predominate over hydrogen bonding among non‐covalent interactions. To delineate the Schiff base contribution, an imine‐free control, BBR@MPN(OCH_3_), was prepared by substituting PEG‐CHO with methoxy‐PEG. Figure S2 showed this control was markedly more vulnerable, with NaCl inducing a ∼tenfold size increase at 50 mm (∼4000 nm vs. ∼1800 nm at 200 mm for BBR@MPN‐NGs) and SDS enlarging particles to ∼2400 nm at 200 mM (vs. ∼400 nm), while urea remained negligible. These data indicated that the Schiff base network acted as a structural lock that raised the disruption threshold. ATR‐FTIR spectroscopy further revealed the underlying molecular interactions (Figure S3). Pure TA displayed characteristic peaks at 1310, 1179, and 1019 cm^−1^, assigned to C‐O stretching, hydroxyl bending, and C‐H bending vibrations, respectively [48]. In BBR@MPN‐NGs, these peaks shifted to 1327, 1189, and 1032 cm^−1^, confirming the coordination between TA and Zn^2+^. To verify Schiff base formation, the ^1^H NMR spectrum of BBR@MPN‐NGs exhibited a new peak at 8.87 ppm that was absent in both PEG‐CHO and BBR@MPN(OCH_3_) controls (Figure 2G). This signal corresponded to the imine proton of the Schiff base moiety, while the concomitant decrease in the aldehyde proton signal confirmed the reaction between PEG‐CHO and CMCS. ATR‐FTIR spectra of BBR@MPN‐NGs showed a new absorption band at 1570 cm^−1^, attributed to the imine bond (C═N) stretching vibration of the Schiff base (Figure 2H and Figure S4) [49, 50]. Consistent with these findings, XPS analysis revealed deconvoluted C 1s and N 1s peaks at 286.9 and 400.2 eV, respectively, both assigned to the C═N bond [51, 52, 53], further confirming the formation of Schiff base linkages (Figure 2J,K).

Drug loading and encapsulation efficiencies were subsequently determined. The BBR loading efficiency was 4.11 ± 0.2%, and the encapsulation efficiency reached 81.32 ± 0.38% (Figure S5). For TA and Zn quantification, the Zn^2^^+^ loading efficiency was 1.09 ± 0.15%, the TA loading efficiency was 45.29 ± 4.37%, and the synthetic yield was 32.4%. Notably, the maximum BBR loading was limited by colloidal stability, as particle size and distribution deteriorated when the TA:BBR feeding ratio exceeded 1:0.5 (Figure 2E). To mimic the physiological environment of the ocular surface, in vitro drug release was evaluated in simulated tear fluid containing the principal electrolytes and proteins of tears (Figure 2I). The result demonstrated that BBR@MPN‐NGs enabled sustained release of BBR throughout the dosing interval.

Collectively, the synergistic integration of dynamic Schiff base chemistry with metal‐phenolic coordination yielded structurally robust dual‐dynamically crosslinked nanogels. This optimized system achieved efficient BBR loading and maintained consistent release profiles in the presence of physiological proteins such as lysozyme, highlighting its potential for DED therapy.

### 2.2 Stability of BBR@MPN‐NGs

Building on the confirmed dual‐dynamically crosslinked architecture, we next evaluated the colloidal stability of the nanogels. BBR@MPN‐NGs aggregated in salt‐containing media lacking surfactant and remained stable in the Tween 80‐containing vehicle (Figure S6). Long‐term durability was assessed by storing the nanogels at 4 °C in the dark for 1 year. No visible aggregation or precipitation was observed, and the PDI remained consistently below 0.1 (Figure S7), demonstrating exceptional long‐term colloidal stability. TEM images further confirmed that the nanogels retained their intact spherical morphology throughout the storage period (Figure S8A). The pharmaceutical integrity was likewise preserved, with the TA content and BBR retention both remaining above 90% of their respective initial values over the 12‐month period, corresponding to less than 10% drug leakage (Figure S8B,C), and the dispersion pH stayed stable at approximately 6 (Figure S8F). For experimental use, the stored nanogels were sampled under aseptic conditions and diluted to the working concentration with phosphate‐buffered saline (PBS) containing 0.1% Tween 80. Together, these data demonstrate that the formulation possesses one‐year stability, markedly exceeding that of most pristine metal‐phenolic nanoparticles, which are inherently unstable in aqueous solution [43]. Such enhanced stability arises not only from the robust structural stability conferred by the dual‐dynamically crosslinked architecture, but also from the unique topological characteristics of PEG‐CHO. Specifically, covalent crosslinking of PEG‐CHO with CMCS simultaneously generates cyclic PEG topologies. Owing to their conformational confinement, these cyclic polymers promote denser chain packing at the particle interface, thereby yielding significantly greater steric hindrance than that of linear counterparts [44, 54]. Such exceptional stability is highly advantageous for biomedical applications.

### 2.3 Free Radical Scavenging and Lubrication Properties of BBR@MPN‐NGs

Given the pronounced antioxidant activity of TA, the 1,1‐diphenyl‐2‐picrylhydrazyl radical (DPPH) and 2,2'‐Azino‐bis(3‐ethylbenzothiazoline‐6‐sulfonic acid) (ABTS) radical scavenging capacities of BBR@MPN‑NGs were systematically evaluated (Figure 3A,B). The scavenging rates increased with TA concentration, reaching 93.52 ± 0.17% for DPPH and 98.10 ± 0.30% for ABTS at 10 µg mL^−1^. Crucially, after one year of storage, the nanogels retained their antioxidant activity, with scavenging rates of 93.27 ± 0.25% for DPPH and 95.93 ± 0.96% for ABTS (Figure S8D,E), confirming the long‑term durability of their antioxidant activity.

**FIGURE 3:**
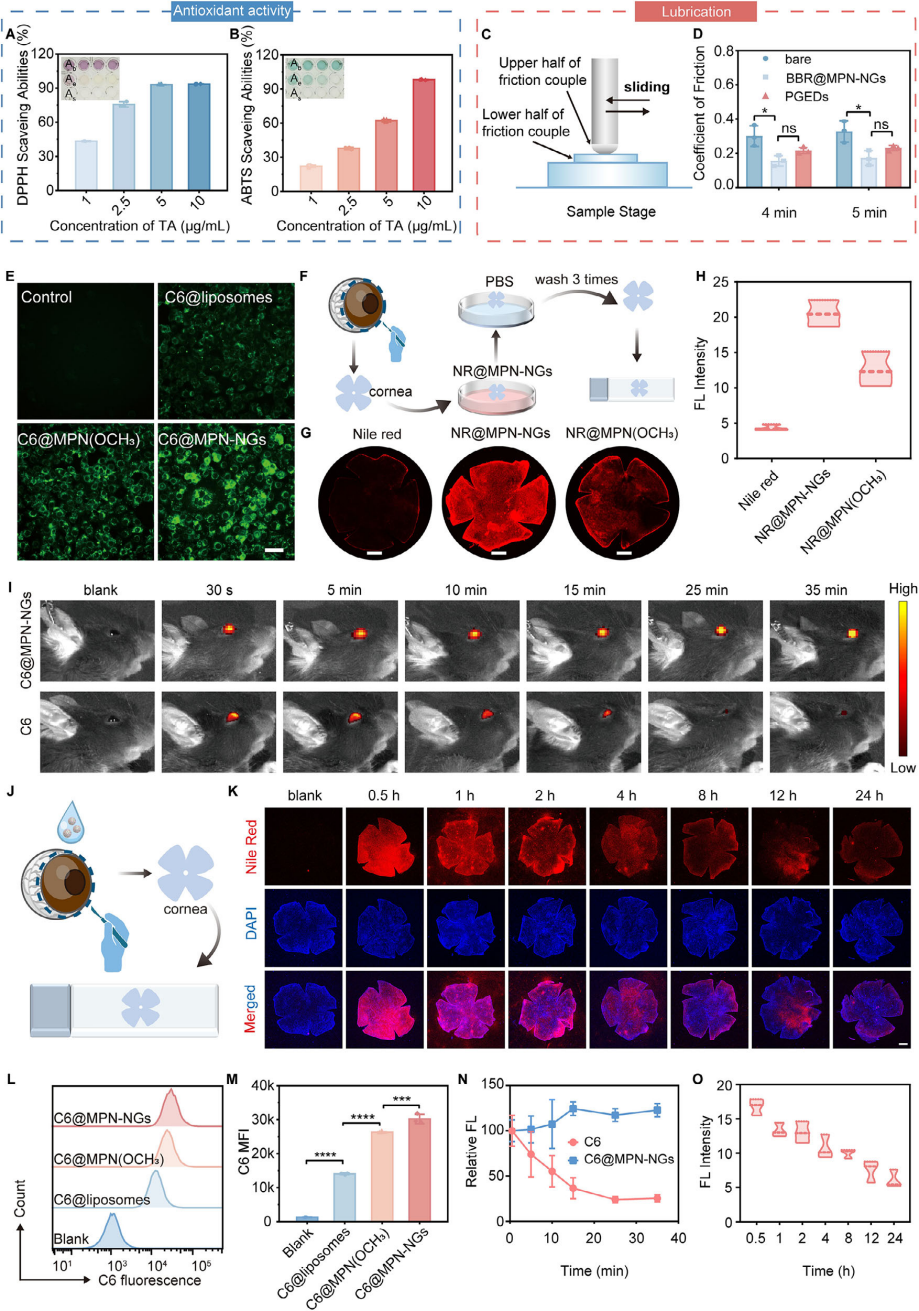
Antioxidant activity, lubrication, enhanced bioadhesion, and prolonged precorneal retention of the nanogels. (A, B) DPPH (A) and ABTS (B) radical scavenging rates of freshly prepared BBR@MPN‐NGs. Inserts: photographs of solutions after different treatments. (C) Schematic of the lubrication measurement. (D) COF of bare, PGEDs‐coated, and BBR@MPN‐NGs‐coated silicon wafers. (E) The fluorescence images of HCECs treated with C6@liposomes, C6@MPN(OCH_3_), or C6@MPN‐NGs. Scale bar: 50 µm. (F) Experimental procedure for assessing rapid adhesion of MPN‐NGs to corneal tissue. (G, H) Confocal images (G) and semiquantitative analysis (H) of Nile red fluorescence on corneal flat‐mounts after incubation with free Nile red, NR@MPN‐NGs, or NR@MPN(OCH_3_) for 10 s. Scale bar: 500 µm. (I) Real‐time in vivo fluorescence images of mouse eyes after instillation of free C6 or C6@MPN‐NGs. (J) Experimental procedure for evaluating corneal retention after topical instillation of NR@MPN‐NGs. (K) Confocal images of corneal flat‐mounts collected at various time points after NR@MPN‐NG application. Scale bar: 500 µm. (L) Flow cytometry analysis of C6 fluorescence in HCECs treated with C6@liposomes, C6@MPN(OCH_3_), or C6@MPN‐NGs for 1 min. (M) Quantitative analysis of mean fluorescence intensity from (L). (N) Quantitative analysis of time‑dependent fluorescence intensity changes in mouse eyes after administration of free C6 or C6@MPN‐NGs. (O) Semiquantitative analysis of Nile red fluorescence on corneal flat‐mounts at different time points after NR@MPN‐NG application. Data are presented as the mean ± SD (*n* = 3). Statistical significance was determined using one‐way ANOVA (ns, *p* > 0.05, ^*^p < 0.05, ^***^p < 0.001, ^****^p < 0.0001). Illustrations in C, F, and J were created using Adobe Illustrator.

In addition to its antioxidant activity, effective lubrication is essential for relieving dry eye discomfort, as it helps restore tear film osmolarity and thereby alleviates hyperosmotic stress on corneal cells [55, 56]. The lubricating properties of BBR@MPN‐NGs were subsequently characterized (Figure 3C). The coefficient of friction (COF) of bare silicon wafers remained steady at ∼0.3 throughout extended sliding cycles (Figure 3D). A commercial polyethylene glycol eye drop (PGEDs) reduced the COF to ∼0.2, and BBR@MPN‐NGs achieved a comparable COF of ∼0.16. The COF of both formulations was significantly lower than that of the bare substrate, indicating that the nanogel exhibits promising in vitro lubricating properties attributable to the CMCS component.

### 2.4 Enhanced Bio‐Adhesion and Protracted Precorneal Retention of Nanogels

Efficient drug accumulation at pathological sites is a prerequisite for therapeutic success, necessitating ocular delivery vehicles with robust adhesion and prolonged surface retention. To this end, we systematically evaluated the adhesive and surface retention properties of the nanogels through a combination of in vitro and in vivo assays.

Hydrophobic coumarin 6 (C6) was encapsulated to yield C6@MPN‐NGs (∼200 nm, Figure S9). Compared with C6‐loaded liposomes (C6@liposomes) and Schiff base‐deficient controls (C6@MPN(OCH_3_)), C6@MPN‑NGs displayed markedly enhanced membrane‑associated fluorescence on human corneal epithelial cells (HCECs) after only 1 min of incubation (Figure 3E). Flow cytometry confirmed these observations, with C6@MPN‑NG‑treated cells exhibiting the highest fluorescence intensity (Figure 3L,M). These results indicated that MPN‐NGs possessed superior cellular adhesion, prompting subsequent assessment of their rapid adhesion to corneal tissue. Given its superior photostability, Nile Red (NR) was encapsulated into the nanogels (NR@MPN‐NGs, Figure S10) for this purpose. Ex vivo mouse corneas exposed to NR@MPN‐NGs for 2 s retained detectable signals even after rigorous washing, and the signal intensity increased progressively up to 20 s (Figure 3F and Figure S11). Notably, after a 10 s application, NR@MPN‐NGs showed significantly stronger corneal adhesion than free NR or Schiff base‐deficient controls (NR@MPN(OCH_3_)) (Figure 3G,H). These results confirmed that the combination of dynamic Schiff base chemistry and TA‐mediated adhesion endowed MPN‐NGs with rapid and robust ocular surface anchoring capabilities.

To validate the ocular surface retention conferred by this corneal adhesion, real‐time in vivo fluorescence imaging was employed to monitor the distribution and persistence of the nanogels in mouse eyes. As shown in Figure 3I, the fluorescence signal of free C6 declined continuously within 30 s after instillation, whereas the C6@MPN‑NGs group maintained a stable and intense signal. Notably, the fluorescence intensity in the C6@MPN‑NGs group increased to 122.7% of its initial value by 35 min post‐administration, while that of the free dye dropped to 25.4% (Figure 3N). This signal enhancement is presumed to result from the rapid tear evaporation under the in vivo imaging conditions (37 °C), coupled with reduced tear secretion under anesthesia, leading to the concentration of fluorescent dyes [57]. To exclude this confounding factor, precorneal retention was assessed in non‐anesthetized mice via corneal flat‐mounts (Figure 3J). Given the superior photostability of Nile Red, the fluorescence signal of NR@MPN‐NGs was detectable on the corneal surface for up to 24 h post‐administration (Figure 3K,O). To obtain direct pharmacokinetic evidence for this prolonged retention, BBR levels in corneal tissue and tear fluid were quantified by LC‐MS/MS over the 24 h dosing interval (Figure S12). Following a single topical instillation of BBR@MPN‐NGs, the corneal BBR concentration reached 13.68 µg g^−1^ at 0.5 h and remained at approximately 1.78 µg g^−1^ at 24 h, whereas free BBR was largely cleared within a few hours. Tear BBR concentrations peaked shortly after instillation (42.6 µg mL^−1^ at 0.5 h) and declined rapidly within 2 h, reflecting rapid washout of the non‐adherent fraction, while corneal exposure persisted throughout the dosing interval. The nanogel formulation therefore sustained corneal BBR exposure across the entire dosing interval, providing direct pharmacokinetic support for the once‐daily regimen. These results confirmed that the synergistic interplay of dynamic Schiff base chemistry and TA‐mediated adhesion enabled long ocular surface retention.

### 2.5 In Vitro ROS‐Scavenging and Anti‐Inflammatory Activities of BBR@MPN‐NGs

The pathogenesis of DED involves a pathological interplay between corneal epithelial cells and macrophages. As frontline sensors of the innate immune system, corneal epithelial cells respond to hyperosmolar and oxidative stress by releasing inflammatory mediators that recruit and activate macrophages. Once activated, these key antigen‐presenting cells exhibit functional heterogeneity, wherein classically activated M1 macrophages adopt a strongly pro‐inflammatory phenotype, exacerbating inflammation via cytokine secretion and triggering adaptive immunity. This establishes a self‐perpetuating vicious cycle in which epithelial damage activates macrophages, driving inflammatory responses that further exacerbate epithelial injury, barrier dysfunction, and apoptosis [58, 59]. Thus, both cells represent promising therapeutic targets for DED, and modulating the underlying intracellular signaling pathways may alleviate DED‐associated cell death and corneal tissue damage.

The cellular uptake of C6@MPN‑NGs was first investigated in both HCECs and RAW264.7 macrophages. After incubation, time‑dependent intracellular fluorescence was observed in both cell lines, with prominent cytoplasmic accumulation evident within 15 min (Figures 4A and 5A). Colocalization with LysoTracker Red DND‑99 indicated that the nanogels accumulated in lysosomes, suggesting uptake via the endocytic pathway. Flow cytometry further confirmed the time‑dependent internalization kinetics in both HCECs and RAW264.7 cells (Figure 4B,C and Figure 5B, C). Collectively, these results demonstrated that C6@MPN‑NGs combined robust adhesion with rapid cellular internalization, representing key prerequisites for achieving efficient intracellular delivery and therapeutic efficacy in DED‐relevant cells.

**FIGURE 4:**
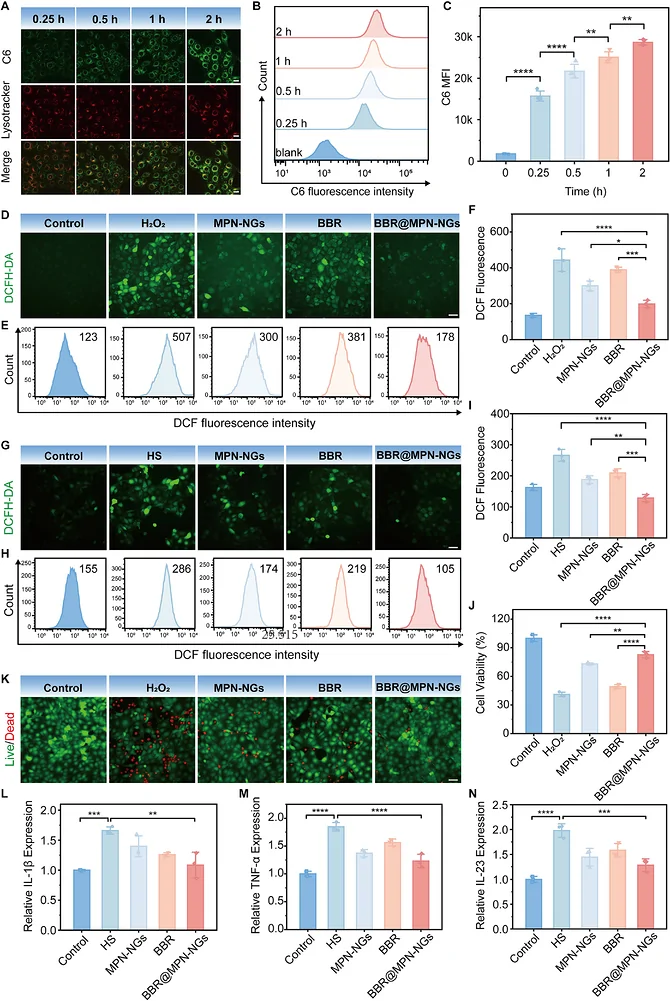
Cellular internalization, antioxidant, anti‐inflammatory, and anti‐apoptotic effects of BBR@MPN‐NGs in HCECs. (A) Representative fluorescence images showing time‐dependent cellular uptake of C6@MPN‐NGs. Green: C6 signal; Red: LysoTracker Red DND‐99. Scale bar: 20 µm. (B) Flow cytometric analysis of C6@MPN‐NGs internalization in HCECs after 0.25, 0.5, 1, and 2 h. (C) Quantitative analysis of fluorescence intensity from (B). (D) Representative fluorescence images of ROS in H_2_O_2_‐stimulated HCECs after the indicated treatments. Scale bar: 50 µm. (E) Flow cytometric analysis of DCF fluorescence in H_2_O_2_‐stimulated HCECs. (F) Quantitative analysis of mean DCF fluorescence intensity from (E). (G) Representative ROS images of HCECs exposed to hyperosmotic stress (HS) and the indicated treatments. Scale bar: 50 µm. (H) Flow cytometric analysis of DCF fluorescence in HCECs under HS. (I) Quantitative analysis of mean DCF fluorescence intensity from (H). (J) Cell viability of HCECs after H_2_O_2_ stimulation and the indicated treatments. (K) Representative Live/Dead staining images of HCECs under H_2_O_2_ induction and the indicated treatments. Scale bar: 50 µm. (L‐N) qPCR analysis of mRNA levels of IL‐1β (L), TNF‐α (M), and IL‐23 (N) in HCECs exposed to HS and the indicated treatments. All data were presented as the mean ± SD (*n* = 3). Statistical significance was determined using one‐way ANOVA (^*^p < 0.05, ^**^p < 0.01, ^***^p < 0.001, ^****^p < 0.0001).

**FIGURE 5:**
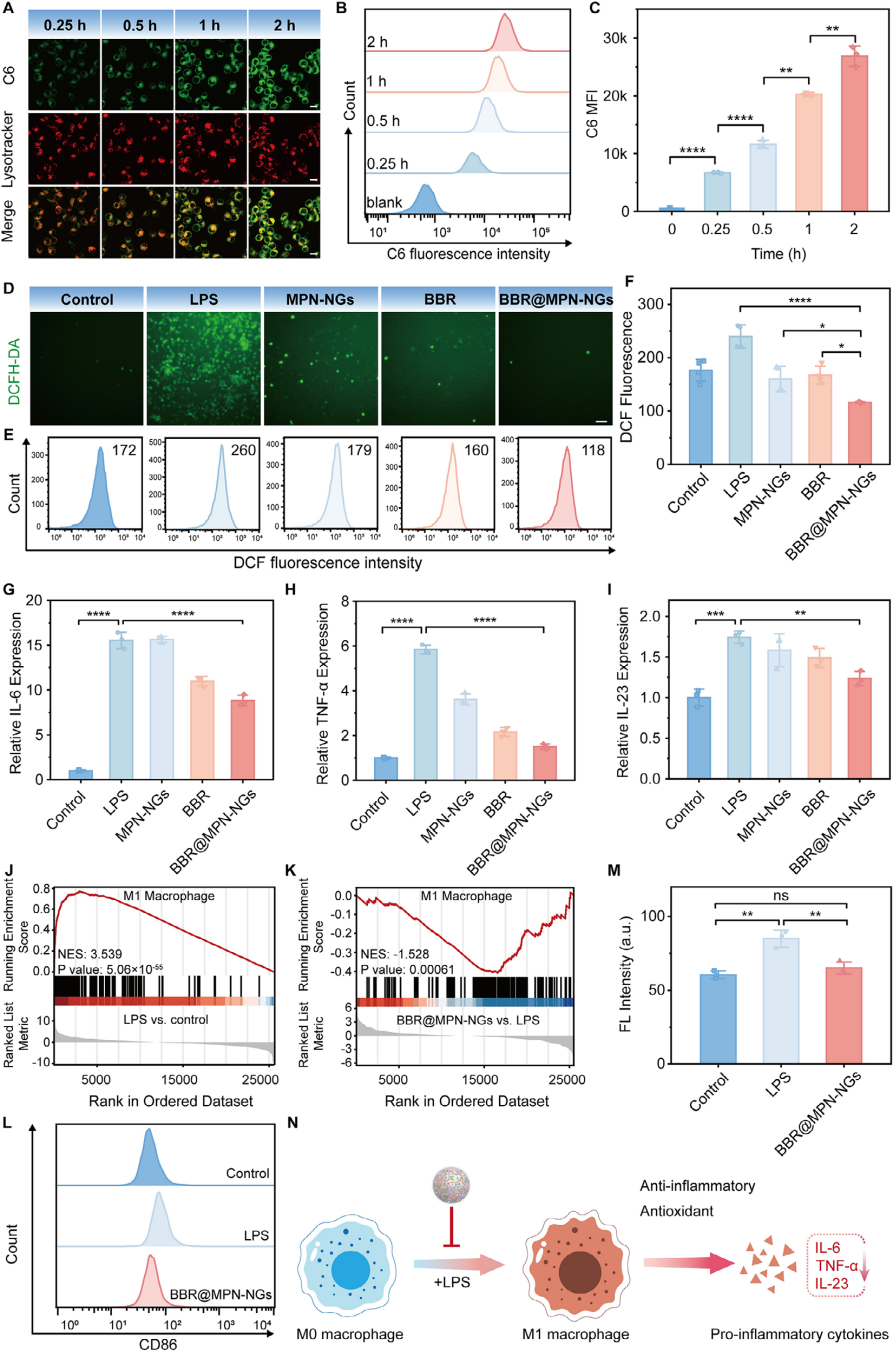
Cellular internalization, antioxidant, anti‐inflammatory, and inhibition of M1 macrophage polarization by BBR@MPN‐NGs in RAW264.7 cells. (A) Representative fluorescence images showing time‐dependent cellular uptake of C6@MPN‐NGs. Green: C6 signal; Red: LysoTracker Red DND‐99. Scale bar: 20 µm. (B) Flow cytometric curves of C6@MPN‐NG internalization in RAW264.7 cells after 0.25, 0.5, 1, and 2 h. (C) Quantitative analysis of fluorescence intensity from (B). (D) Representative fluorescence images of ROS in LPS‐stimulated RAW264.7 cells after the indicated treatments. Scale bar: 50 µm. (E) Flow cytometric analysis of DCF fluorescence in RAW264.7 cells under LPS stimulation with the indicated treatments. (F) Quantitative analysis of mean DCF fluorescence intensity from (E). (G–I) qPCR analysis of mRNA levels of IL‐6 (G), TNF‐α (H), and IL‐23 (I) in LPS‐induced RAW264.7 cells after the indicated treatments. (J) Gene set enrichment analysis (GSEA) comparing LPS‐treated macrophages vs. control in bulk RNA‐seq data. (K) GSEA enrichment analysis comparing LPS + BBR@MPN‐NGs vs. LPS‐treated macrophages in bulk RNA‐seq data. (L) Flow cytometric curves of CD86 expression in LPS‐stimulated RAW264.7 cells after the indicated treatments. (M) Quantitative analysis of CD86‐positive cells from (L). (N) Schematic illustration showing inhibition of M1 macrophage polarization by BBR@MPN‐NGs in RAW264.7 cells. All data were presented as the mean ± SD (*n* = 3). Statistical significance was determined using one‐way ANOVA (ns, *p* > 0.05, ^*^p < 0.05, ^**^p < 0.01, ^***^p < 0.001, ^****^p < 0.0001). Illustration in N was created using Adobe Illustrator.

Given the pathological role of ROS in driving inflammation and cytokine secretion in DED, the ROS‑scavenging efficacy of BBR@MPN‑NGs was evaluated. Hydrogen peroxide (H_2_O_2_) was used to induce oxidative stress in HCECs. Intracellular ROS levels were monitored with the fluorescent probe 2′,7′‑dichlorofluorescein diacetate (DCFH‑DA). BBR@MPN‑NGs suppressed ROS generation most effectively, whereas blank MPN‑NGs and free BBR provided only moderate suppression, as visualized by fluorescence microscopy (Figure 4D) and quantified by flow cytometry (Figure 4E,F). To further mimic the tear hyperosmolarity characteristic of DED, HCECs were exposed to hypertonic medium (450 mOsm L^−1^). Consistent with the results obtained under H_2_O_2_ challenge, BBR@MPN‑NGs significantly attenuated hyperosmolarity‑induced ROS production (Figure 4G–I). The evaluation was then extended to immune cells by stimulating RAW264.7 macrophages with lipopolysaccharide (LPS). While blank MPN‐NGs and free BBR partially mitigated LPS‑induced ROS generation, BBR@MPN‑NGs exhibited superior scavenging efficiency (Figure 5D–F). These results underscored the enhanced antioxidant capability of BBR@MPN‑NGs across both epithelial and immune cell populations of the ocular surface.

The anti‐inflammatory efficacy of the formulations was subsequently evaluated by quantitative real‐time polymerase chain reaction (qPCR). Under hyperosmotic stress (HS) stimulation, HCECs exhibited 1.66‑, 1.85‑, and 1.98‑fold increases in the mRNA levels of IL‐1β, TNF‐α, and IL‐23, respectively (Figure 4L–N). While blank MPN‑NGs and free BBR moderately attenuated these increases, BBR@MPN‑NGs demonstrated superior efficacy, reducing the expression levels to 1.09‑, 1.23‑, and 1.29‑fold relative to the control. To delineate the contribution of each nanogel component, the individual constituents and their combinations were evaluated in the same HS‐stimulated HCEC model (Figure S13). TA partially suppressed hyperosmolarity‐induced TNF‐α and IL‐23 transcription, while Zn^2^^+^ and CMCS individually conferred moderate suppression of TNF‐α. Consistently, TA produced the most pronounced reduction in intracellular ROS levels, as quantified by DCF fluorescence intensity and confirmed by fluorescence microscopy, whereas the Schiff base network formed by CMCS and PEG‐CHO exhibited no detectable anti‐inflammatory or antioxidant activity. These experiments established a clear division of labor within the nanogel, in which TA is the principal contributor to the antioxidant activity, the anti‐inflammatory effect is dominated by BBR and TA with auxiliary contributions from Zn^2^^+^ and CMCS, and the Schiff base network serves primarily a structural role in maintaining colloidal stability and ocular retention. Similarly, in LPS‑stimulated RAW264.7 macrophages, BBR@MPN‑NGs achieved the most pronounced suppression of inflammatory cytokines, outperforming blank MPN‐NGs and free BBR (Figure 5G–I). To further explore the immunomodulatory effects, macrophage polarization was assessed by flow cytometry using the M1 marker CD86 (Figure 5L,M). LPS markedly elevated the proportion of CD86‐positive M1 macrophages, whereas BBR@MPN‑NGs substantially inhibited this pro‐inflammatory polarization, restoring the M1 proportion to near‐baseline levels. Collectively, these results demonstrate that BBR@MPN‐NGs effectively suppress the expression of key inflammatory cytokines and inhibit M1 macrophage polarization (Figure 5N).

The therapeutic potential of BBR@MPN‐NGs against oxidative stress‑induced cell death, a hallmark of DED, was next evaluated. Live/Dead staining revealed that BBR@MPN‑NGs significantly reduced H_2_O_2_‐induced cytotoxicity (Figure 4K), a result further supported by 3‐(4,5‐dimethylthiazol‐2‐yl)‐2,5‐diphenyltetrazolium bromide (MTT) assays. H_2_O_2_ exposure decreased HCEC viability to 41.07 ± 2.35% relative to the untreated control (Figure 4J). In contrast, blank MPN‑NGs restored viability to 73.03 ± 1.18%, while BBR@MPN‑NGs afforded superior protection, raising viability to 82.69 ± 3.29%. Notably, the substantial protection conferred by blank MPN‐NGs alone indicated that the TA‐based carrier itself contributes markedly to cytoprotection. To verify that the selected TA:BBR ratio represents a functional balance rather than a loading limitation, nanogels prepared at ratios of 1:0.25 and 1:0.5 were compared at matched BBR concentrations (Figure S14). The TA‐richer formulation (1:0.25, TA 8.5–17 µg mL^−1^) provided higher cell viability than the BBR‐richer formulation (1:0.5, TA 4.9‐9.8 µg mL^−1^), with the difference reaching statistical significance at 2.5 µm BBR. This confirmed that increasing the BBR fraction necessarily diluted TA and diminished its antioxidant contribution to overall efficacy. These results indicated that BBR@MPN‑NGs effectively protect corneal epithelial cells from oxidative stress‐mediated cell death, underscoring their potential as a multimodal therapeutic platform for DED.

To elucidate the underlying intracellular mechanisms, whole‑transcriptome RNA sequencing was performed on both HCECs and RAW264.7 cells. In HCECs, 1956 genes upregulated by hyperosmotic stress were significantly downregulated upon BBR@MPN‑NG treatment (Figure 6A–C). Heatmap analysis revealed that BBR@MPN‐NGs specifically suppressed genes associated with inflammation, chemotaxis, and oxidative stress (e.g., *TNF, MMP‐10, PTGS2*), while upregulating antioxidant (e.g., *NFE2L3, GPX8*) and mucin‐promotion transcripts such as *MUC4* (Figure 6D). Kyoto Encyclopedia of Genes and Genomes (KEGG) and Gene Ontology (GO) pathway enrichment analyses identified the MAPK signaling pathway, particularly the JNK cascade, as a key target (Figure 6E,F). Western blotting further confirmed that BBR@MPN‑NGs markedly suppressed HS‑induced JNK phosphorylation (Figure 6G and Figure S15), validating these bioinformatic predictions. To further investigate the functional involvement of JNK/MAPK signaling in the anti‐inflammatory effects of BBR@MPN‐NGs, a pharmacological rescue experiment was performed using anisomycin, an activator of the p38/JNK pathway (Figure S16). Co‐incubation with anisomycin abolished the nanogel‐mediated suppression of IL‐23, restoring its transcription to a level comparable to the hyperosmolarity group, and partially rescued the suppression of TNF‐α, demonstrating that the anti‐inflammatory effect of BBR@MPN‐NGs is mediated primarily through inhibition of the JNK/MAPK pathway. Similarly, in RAW264.7 cells, 454 genes upregulated by LPS stimulation were downregulated after treatment with BBR@MPN‐NGs (Figure S17). Heatmap analysis further demonstrated that BBR@MPN‑NGs reduced pro‐inflammatory and oxidative stress gene signatures while enhancing anti‐inflammatory and antioxidant transcripts (Figure S18). GSEA analysis confirmed that BBR@MPN‑NGs significantly reversed the gene signature of LPS‑induced M1 polarization (Figure 5J,K), consistent with flow cytometry results.

**FIGURE 6:**
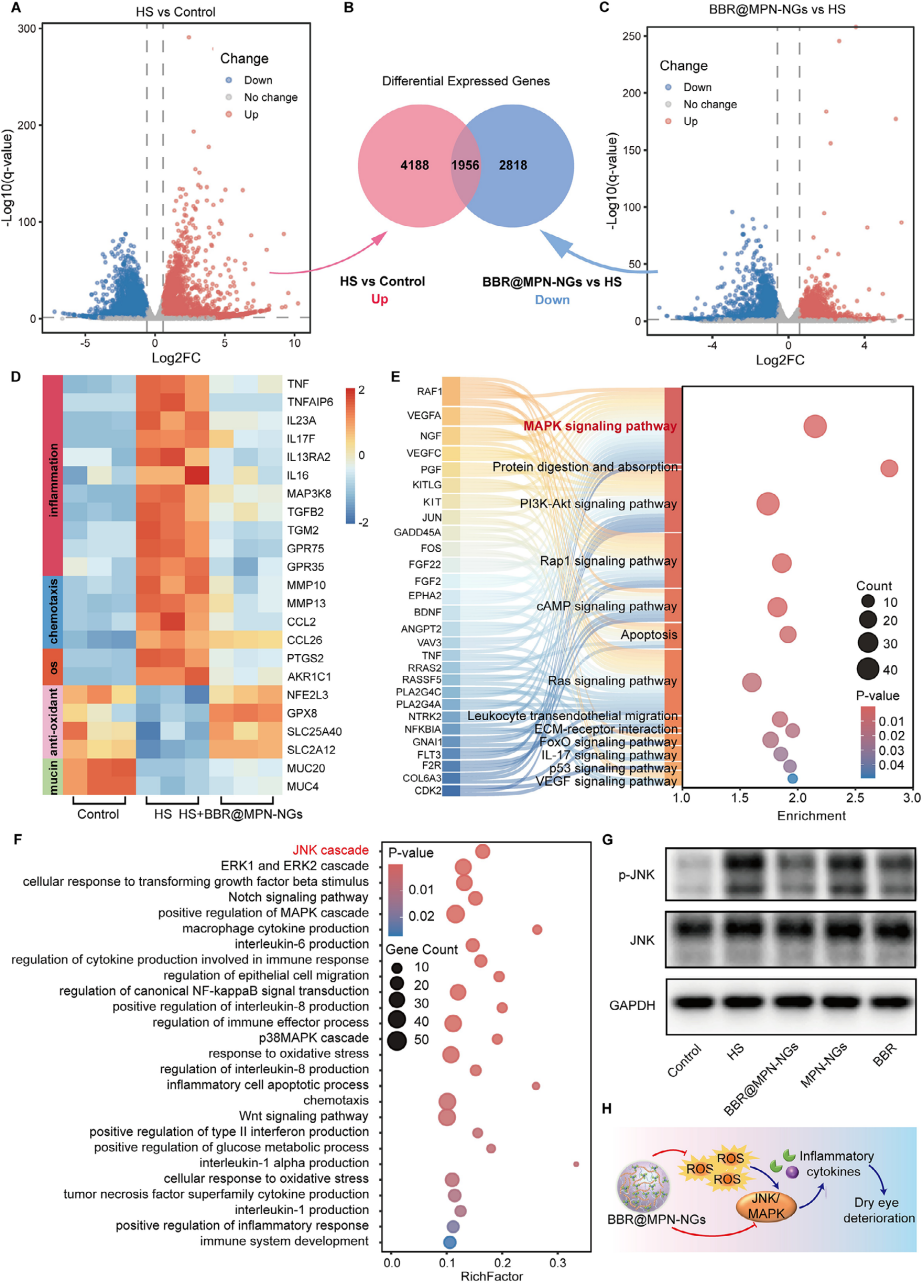
RNA‐seq and western blot analyses of HCECs treated with BBR@MPN‐NGs. (A) Volcano plot showing differentially expressed genes between the HS and control groups. (B) Venn diagram depicting the overlap of genes significantly upregulated in the HS group and significantly downregulated in the BBR@MPN‑NGs group. (C) Volcano plot comparing the HS + BBR@MPN‐NGs and HS groups. (D) Heatmap of significantly upregulated and downregulated genes in HCECs after BBR@MPN‐NGs treatment (fold change ≥1.5, p < 0.05). (E) KEGG pathway enrichment analysis of differentially expressed genes between the HS + BBR@MPN‐NGs and HS groups. (F) GO enrichment analysis of differentially expressed genes between the HS + BBR@MPN‐NGs and HS groups. (G) Western blot images showing protein levels of p‐JNK, JNK, and GAPDH in HCECs stimulated with HS (450 mOsM L^−1^) for 2 h. (H) Schematic illustration of the proposed mechanism by which BBR@MPN‐NGs inhibit the JNK/MAPK signaling pathway. Illustration in H was created using Adobe Illustrator.

Overall, in vitro pharmacological evaluation demonstrates that BBR@MPN‑NGs concurrently suppress ROS generation, downregulate inflammatory and chemotactic factor expression, inhibit macrophage M1 polarization, and effectively attenuate corneal epithelial cell death. Mechanistically, these effects are primarily mediated through suppression of the JNK/MAPK signalling cascade, thereby attenuating a broad spectrum of inflammatory and oxidative stress pathways. These findings establish BBR@MPN‑NGs as a multimodal nanoplatform capable of addressing multiple pathological processes in DED and provide a strong rationale for subsequent in vivo efficacy studies.

### 2.6 BBR@MPN‐NGs for Treatment of BAC‐Induced DED Mouse Models

Given the pivotal roles of oxidative stress, inflammation, and tear film instability in DED pathogenesis, and the demonstrated ROS‐scavenging, anti‐inflammatory, lubricating, and corneal adhesion properties of BBR@MPN‐NGs, we evaluated their therapeutic efficacy as eye drops in a mouse model of DED. The model was established using BAC, a quaternary ammonium compound that disrupts tight junctions between superficial corneal epithelial cells, thereby compromising epithelial barrier function, accelerating tear film breakup and evaporation, and ultimately inducing dry eye symptoms [8]. This approach recapitulates the key clinical manifestations and histopathological changes of human DED, including reduced tear film stability, ocular surface inflammation, corneal epithelial damage, and goblet cell loss.

Mice received topical instillation of 0.2% benzalkonium chloride for seven consecutive days to establish the DED model (Figure 7A). In vivo ocular surface lubrication and comfort were next assessed by monitoring spontaneous blink frequency for 5 min after treatment, using commercial eye drops PGEDs as a positive clinical control. In healthy mice, blink frequency was unaffected by PBS, PGEDs, or BBR@MPN‑NGs, confirming the biocompatibility and non‐irritating nature of the formulations (Figure 7K). In contrast, DED mice exhibited an elevated blink frequency (31.5 ± 4.23 blinks per 5 min) that was markedly reduced to 6 ± 2.53 blinks by BBR@MPN‑NGs, a level comparable to that achieved with PGEDs (8.67 ± 2.50 blinks) (Figure 7L). These results demonstrated that BBR@MPN‑NGs provided ocular surface lubrication and comfort comparable to that of commercial artificial tears.

**FIGURE 7:**
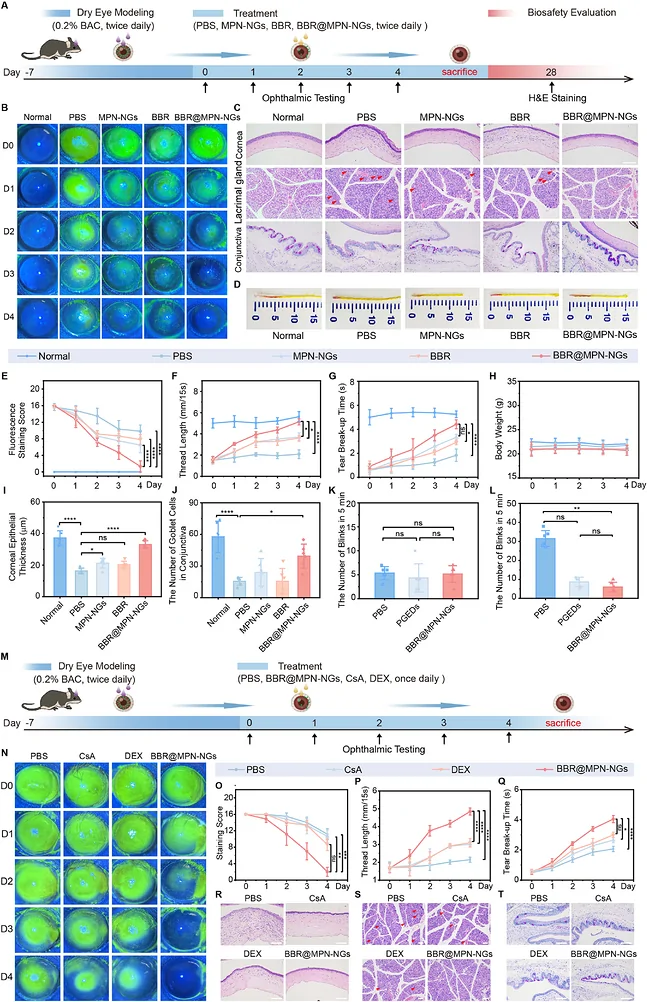
In vivo therapeutic efficacy, reduced dosing frequency, and comparison with clinically relevant eye drops. (A) Schematic of the BAC‐induced DED model and treatment regimen. (B) Representative fluorescein sodium staining images of DED mice over the 4‐day treatment period. (C) Representative H&E staining images of the cornea (upper), lacrimal gland (middle), and PAS staining images of the conjunctiva (lower) in different groups. Scale bar: 100 µm. (D) Representative images of wetted phenol red cotton thread lengths. (E) Corresponding corneal fluorescein staining scores. (F) Tear volume measured by the phenol red thread test. (G, H) TBUT (G) and daily body weight (H) records of the DED mice treated with different therapies. (I) Quantitative analysis of the corneal epithelial thickness. (J) Quantitative analysis of the goblet cell numbers. (K, L) Blink frequency (blinks per 5 min) in healthy mice (K) and DED mice (L) after topical instillation of the indicated formulations. (M) Schematic illustration of the experimental design for evaluating once‐daily dosing of BBR@MPN‐NGs and comparison with CsA and DEX. (N) Representative fluorescein sodium staining images of DED mice treated with BBR@MPN‐NGs, CsA, and DEX over the 4‐day treatment. (O) Corresponding fluorescein staining scores. (P) Tear volume measured by the phenol red thread test. (Q) TBUT of the DED mice treated with different therapies. (R‐T) Representative H&E staining images of the cornea (R), lacrimal gland (S), and PAS staining images of the conjunctiva (T) after 4 days of once‑daily dosing. Scale bar: 100 µm. All data were presented as the mean ± SD (*n* = 6). Statistical significance was determined by one‐way ANOVA, except for panels G, L, O, and Q, which were analyzed by the Kruskal‐Wallis test (ns, *p* > 0.05, ^*^ *p* < 0.05, ^**^ *p* < 0.01, ^***^ *p* < 0.001, ^****^ *p* < 0.0001). Illustrations in A and M were created using Adobe Illustrator.

Following model establishment, mice received topical instillation of PBS, MPN‐NGs, free BBR, or BBR@MPN‐NGs twice daily for 4 consecutive days. Corneal epithelial integrity was assessed by fluorescein staining and quantified using a standardized 16‑point scoring system. Healthy mice maintained minimal corneal fluorescence (Figure 7B,E). In contrast, PBS‑treated DED mice exhibited persistent epithelial damage and severe dry eye symptoms, with scores consistently exceeding 9. Treatment with MPN‑NGs or free BBR alone partially alleviated these manifestations, yet staining scores remained above 6.0 on day 4. Notably, BBR@MPN‑NGs promoted progressive healing, culminating in a mean fluorescein score of only 1.3 by day 4, demonstrating markedly superior corneal restoration. Tear film stability was further evaluated through tear secretion measurements (phenol red thread test) and tear break‑up time (TBUT). Healthy mice exhibited an average tear volume of 5.62 ± 0.48 mm, whereas PBS‑treated DED mice showed a marked reduction to 2.08 ± 0.28 mm (Figure 7D,F). BBR@MPN‑NGs restored tear volume most effectively (5.13 ± 0.26 mm), approaching physiological levels, and significantly prolonged TBUT to 4.42 ± 0.20 s compared to the PBS group (1.83 ± 0.38 s) (Figure 7G).

The restorative effects of BBR@MPN‑NGs on ocular surface morphology were evaluated by histological analysis using hematoxylin‑eosin (H&E) and periodic acid‑Schiff (PAS) staining. H&E staining revealed that BAC induction disrupted corneal epithelial architecture and reduced epithelial thickness to 16.28 ± 1.84 µm in the PBS group (Figure 7C,I). While blank MPN‑NGs (21.31 ± 3.12 µm) and free BBR (20.2 ± 2.2 µm) partially restored epithelial thickness, BBR@MPN‑NGs achieved the most robust regeneration (33.04 ± 2.34 µm) with regular multilayered cellular organization. Similarly, in the lacrimal glands, BBR@MPN‑NGs effectively reversed BAC‑induced disorganization, acinar atrophy, and external lymphocyte infiltration (red arrows), restoring a near‑normal glandular structure. Consistent with these observations, PAS staining showed that BBR@MPN‑NGs substantially restored goblet cell density in the conjunctiva (Figure 7C,J), counteracting the characteristic depletion seen in DED. These results demonstrated that BBR@MPN‑NGs exerted excellent therapeutic effects by promoting the restoration of the corneal epithelium, lacrimal glands, and mucin‑secreting goblet cells, thereby ameliorating ocular surface pathology.

### 2.7 Dosing Frequency Optimization and Comparison of Efficacy With Clinical Drugs

Encouraged by the superior therapeutic efficacy and 24‐h ocular retention of BBR@MPN‐NGs, we next performed a dose‑frequency optimization study (Figure 7M). In the BAC‐induced DED model, a once‑daily topical regimen was applied and compared with the clinical first‑line agents CsA and DEX. Notably, once‑daily dosing of BBR@MPN‑NGs promoted equally rapid restoration of the defective corneal epithelium as frequent dosing, and achieved faster corneal surface recovery by day 2 compared to clinical comparators (Figure 7N,O). By day 4, corneal fluorescein staining scores in the BBR@MPN‐NGs group recovered to those of healthy controls, matching the efficacy of the twice‑daily regimen. This therapeutic advantage extended to functional recovery, with BBR@MPN‑NGs markedly improving tear secretion from 1.67 ± 0.25 mm to 4.85 ± 0.20 mm and TBUT from 0.5 ± 0.13 s to 4.05 ± 0.23 s (Figure 7P,Q). At the end of the treatment, histological analysis of the eyes and lacrimal glands was performed using H&E and PAS staining (Figure 7R–T). Consistent with observations in the frequent‑dosing DED model, PBS‐treated DED mice exhibited extensive superficial epithelial damage and epithelial thinning, while the stroma showed thickening with inflammation. BBR@MPN‐NGs effectively repaired the corneal epithelium, restoring both its thickness and integrity. H&E staining of the lacrimal glands showed disorganized glandular architecture with irregularly sized and disordered acini in DED mice. In line with these findings, BBR@MPN‑NG treatment achieved the most pronounced lacrimal gland recovery, with uniformly sized and well‑organized acini. Moreover, conjunctival goblet cell density was substantially restored after BBR@MPN‑NG treatment, outperforming both CsA and DEX monotherapies. These findings underscored the nanogels as a highly promising candidate for DED therapy, uniquely enabling convenient once‑daily dosing through prolonged ocular retention.

Beyond these clinical comparators, we further benchmarked BBR@MPN‐NGs against recently reported ocular delivery platforms. Polysaccharide‐based nanogels [22] offer mucoadhesion and biocompatibility but often lack intrinsic bioactivity and require frequent dosing. Similarly, advanced platforms such as a thermo‐responsive FK506 hydrogel [23] and RGD‐modified alginate microparticles for MSC delivery [24] still necessitate twice‐daily administration, with the former showing corneal pharmacokinetics characterized only up to 8 h and the latter limited to 80–120 min of precorneal retention. Even sophisticated nanocarriers co‐loading antioxidants and anti‐inflammatories, such as cationic micelles [57], demonstrate multimodal efficacy but ultimately suffer from rapid precorneal clearance. In contrast, BBR@MPN‐NGs achieve once‐daily dosing supported by quantifiable corneal drug levels at 24 h post‐administration, highlighting the practical advantage of the dual‐dynamic architecture, which integrates antioxidant, anti‐inflammatory, and lubricating functions within a single formulation.

It is worth noting that this therapeutic outcome was achieved at a modest BBR loading of approximately 4%. Because the nanogel carrier itself is pharmacologically active through the antioxidant, anti‐inflammatory, and lubricating functions of TA, Zn^2^^+^, and CMCS, the therapeutic demand on BBR content is intrinsically lower than that for inert carriers. Together with the low micromolar effective concentrations observed in vitro, the superior in vivo efficacy of once‐daily BBR@MPN‐NGs over CsA and DEX supports the adequacy of the present loading for clinical translation.

### 2.8 BBR@MPN‐NGs for Treatment of Scopolamine‐Induced DED Mouse Models

To assess the general applicability of BBR@MPN‐NGs across dry eye etiologies, therapeutic efficacy was further evaluated in a scopolamine‐induced aqueous‐deficient dry eye model, which is mechanistically distinct from the preservative‐driven BAC model (Figure S19A). Following once‐daily topical administration for 5 days, BBR@MPN‐NGs produced the most pronounced corneal epithelial recovery among all treatment groups, with fluorescein staining scores decreasing to approximately 1 by day 5, whereas scores in the PBS, blank MPN‐NGs, free BBR, CsA, and DEX groups remained around 6 to 10 (Figure S19B,C). Functional recovery followed the same trend, with tear secretion increasing to approximately 4.7 mm per 15 s and tear break‐up time prolonging to approximately 4.8 s, both significantly exceeding the other groups (Figure S19D,E). Histological analysis further demonstrated that BBR@MPN‐NGs restored conjunctival goblet cell density and alleviated inflammatory cell infiltration in the lacrimal glands more effectively than CsA and DEX (Figure S19F). The reproducibility of these therapeutic effects in two etiologically distinct models supports the broad applicability of the nanogel platform for dry eye disease.

### 2.9 In Vivo Underlying Mechanisms

To further elucidate the mechanism of dry eye symptom recovery in mice under the intervention of BBR@MPN‐NGs eye drops, immunofluorescence staining was performed on corneal sections. Oxidative stress was assessed using dihydroethidium (DHE), a cell‐permeable probe that fluoresces red upon oxidation by intracellular ROS. DHE staining revealed markedly elevated ROS levels in the corneal epithelium of DED mice (Figure 8A,B). After four days of treatment, MPN‑NGs and free BBR reduced ROS accumulation by 41.82% and 38.19%, respectively. In striking contrast, BBR@MPN‑NGs achieved 82.46% suppression, indicating superior antioxidant activity. The expression of key pro‑inflammatory factors, including IL‑1β, TNF‑α, and MMP‑9, was also evaluated. All were substantially upregulated in DED mice. MPN‑NGs reduced these cytokines by 46.33%, 34.26%, and 58.13%, respectively, whereas free BBR exerted more moderate inhibition (24.19%, 27.83%, and 31.71%). Notably, BBR@MPN‑NGs produced the most potent suppression, with reductions of 60.86%, 62.93%, and 94.33%, respectively (Figure 8C–E).

**FIGURE 8:**
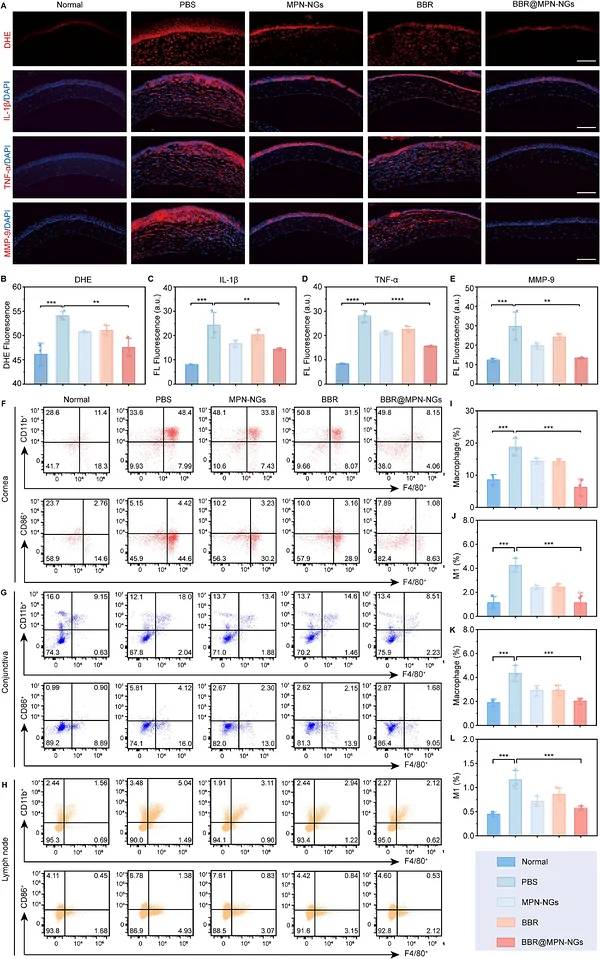
Antioxidant, anti‐inflammatory, and M1 macrophage polarization‐inhibiting effects of BBR@MPN‐NGs in vivo. (A) Representative immunofluorescence images of oxidative stress and inflammatory cytokines IL‐1β, TNF‐α, and MMP‐9 in corneal sections. Scale bar: 100 µm. (B‐E) Quantitative analysis of total fluorescence intensity for DHE (B), IL‐1β (C), TNF‐α (D), and MMP‐9 (E) (n = 3). (F‐H) Flow cytometric analysis of the percentages of the CD11b^+^F4/80^+^ macrophages and CD86^+^F4/80^+^ M1 macrophages in corneas (F), conjunctiva (G), and lymph node (H). (I, J) Quantification of macrophages (I) and M1 macrophages (J) in the conjunctiva. (K, L) Quantification of macrophages (K) and M1 macrophages (L) in the lymph node. Data were presented as mean ± SD. (*n* = 3). Statistical significance was determined using one‐way ANOVA (^**^p < 0.01, ^***^p < 0.001, ^****^p < 0.0001).

Immunophenotypic analysis further revealed the immunomodulatory function of BBR@MPN‑NGs beyond direct ROS scavenging. Flow cytometry was used to quantify the proportions of total macrophages (CD11b^+^F4/80^+^) and M1‑polarized macrophages (CD86^+^F4/80^+^) in the cornea, conjunctiva, and lymph nodes across treatment groups (Figure 8F–L). DED induction significantly elevated both macrophage populations in all three tissues, providing a cellular basis for the upregulated expression of inflammatory mediators. While all interventions reduced macrophage infiltration and M1 polarization, BBR@MPN‑NGs exerted the most potent suppression, restoring these frequencies to levels approaching those of healthy controls. Collectively, BBR@MPN‑NGs integrate antioxidant and anti‑inflammatory functions to suppress ocular surface macrophage infiltration and M1 polarization, thereby promoting ocular surface tissue restoration and representing a promising strategy for the treatment of dry eye disease.

### 2.10 Safety Evaluation of BBR@MPN‐NG Eye Drops

In light of the prominent therapeutic efficacy of BBR@MPN‐NGs, its biosafety profile was rigorously evaluated to ensure compatibility with the delicate ocular microenvironment, a prerequisite for clinical translation. Conventional eye drops often rely on preservatives that can exacerbate dry eye disease through ocular toxicity and disruption of the ocular surface microenvironment. Notably, the nanogels maintain robust colloidal stability for up to one year under preservative‐free conditions. To directly confirm the safety of the instilled preparation, sterility testing and endotoxin detection were performed. The results showed that the nanogel formulation tested negative for microbial contamination under aseptic culture conditions (Figure S20A), and endotoxin levels were below 0.5 EU mL^−1^ as determined by the limulus amebocyte lysate (LAL) gel‐clot method (Figure S20B). From a translational perspective, BBR@MPN‐NGs are prepared by sequential assembly in a predominantly aqueous medium under mild conditions using conventional mixing. Formulation quality was characterized by particle size, polydispersity, BBR encapsulation efficiency, TA content, and pH. The concentrated aqueous dispersion maintained physicochemical stability for up to 12 months when stored at 4°C and protected from light. No microbial growth was observed in the sterility assay, and endotoxin levels in the tested preparation were below 0.5 EU mL^−1^. Together, these preparation characteristics and quality assessments provide an experimental basis for further pharmaceutical development of the preservative‐free ocular formulation.

The cytocompatibility of BBR@MPN‐NGs was subsequently assessed by MTT assay. As shown in Figure S21, no significant cytotoxicity was observed in HCECs or RAW264.7 cells across a range of BBR concentrations (1.25‐10 µM), corresponding to TA concentrations of 4.26‐34.1 µg mL^−1^. In vivo biocompatibility was monitored during the DED treatment study, where all treatment groups (PBS, MPN‐NGs, free BBR, and BBR@MPN‐NGs) maintained stable body weights and normal behavior (Figure 7H). Ocular tolerability was specifically evaluated in healthy mice receiving topical instillation of BBR@MPN‐NGs (5 µL per eye, twice daily) for 28 days. Slit‐lamp examination and corneal fluorescein staining revealed no epithelial defects or signs of irritation (Figure S22A). Histological analysis of the eyeballs, conjunctiva, and lacrimal glands further confirmed the absence of pathological changes, with corneal architecture, epithelial cell arrangement, and goblet cell density remaining indistinguishable from PBS‐treated controls (Figure S22B–E). Given that repeated exposure to zinc‐containing nanomaterials may raise accumulation concerns, Zn levels in ocular surface tissues were quantified by ICP‐MS on days 0, 14, and 28 (Figure S23). The Zn concentration remained unchanged at approximately 9 µg g^−1^ compared with the day 0 baseline, with no apparent increase during repeated exposure, consistent with the low Zn^2+^ loading of the nanogels and the homeostatic regulation of zinc in ocular tissues. Systemic toxicity was assessed by histopathological examination of major organs (heart, liver, spleen, lung, and kidney). H&E staining revealed no overt tissue damage or lesions in BBR@MPN‐NG‐treated mice relative to controls (Figure S24).

Collectively, the preservative‐free nanogels pass sterility and endotoxin testing. Together with the absence of ocular and systemic toxicity, these attributes demonstrate an excellent biocompatibility and safety profile, substantially enhancing the translational potential of this eye drop formulation.

## 3 Conclusion

In summary, we have developed and evaluated a novel BBR‐loaded dual‐dynamically crosslinked nanogel (BBR@MPN‐NGs) that integrates TA‐Zn^2+^ metal‐phenolic networks with CMCS/PEG‐CHO Schiff base networks to enable a single‐nanoplatform multimodal therapy integrating antioxidant, anti‐inflammatory, and lubricating capacities. By unifying these three multimodal functionalities alongside prolonged ocular retention and preservative‐free long‐term stability, this nanoplatform overcomes the long‐standing limitations of existing dry eye therapies, which typically rely on single targets, demand polypharmacy and frequent dosing owing to rapid clearance, and suffer from poor stability, replacing single‐target palliative approaches with integrated multimodal intervention. The dual‐dynamically crosslinked architecture ensures exceptional colloidal stability and antioxidant activity for up to 1 year. The synergy of TA‐mediated wet adhesion and dynamic Schiff base bonds supports sustained corneal BBR exposure for up to 24 h, enabling a convenient once‐daily regimen that matches the efficacy of frequent dosing regimens and outperforms clinical benchmarks, such as CsA and DEX. From a translational perspective, the murine conjunctival sac has a tear volume below 0.2 µL compared with approximately 7 µL in humans, resulting in a markedly higher instilled‐to‐resident volume ratio and more pronounced overflow and initial drainage of the instilled dose [60, 61]. Thus, the 24‐h corneal drug levels and once‐daily efficacy observed in this mouse model provide a preclinical foundation supporting the potential for once‐daily dosing in humans. Future clinical trials will determine the optimal dosing schedule. Mechanistically, BBR@MPN‐NGs reprogram the pathological microenvironment by scavenging ROS, inhibiting pro‐inflammatory macrophage polarization, and downregulating pro‐inflammatory gene expression through blockade of the JNK/MAPK signaling pathway. Notably, the preservative‐free nanogels pass sterility and endotoxin testing, and the absence of ocular and systemic toxicity further supports their biosafety. Collectively, these dual‐dynamically crosslinked nanogels transform dry eye management from frequent palliative care into sustained pathological intervention, reducing dosing frequency and shortening the treatment duration. This nanoplatform provides a high‐compliance therapeutic regimen for chronic DED and a versatile strategy for complex ocular surface disorders requiring sustained retention and multimodal intervention.

## 4 Experimental Section

### 4.1 Materials and Reagents

Berberine (BBR) was obtained from Shanghai Yuanye Biotechnology Co., Ltd (Shanghai, China). Carboxymethyl chitosan (CMCS), DPPH, Coumarin 6 (C6), Nile Red (NR), and 2′,7′‐dichlorofluorescein diacetate (DCFH‐DA) were purchased from Aladdin (Shanghai, China). Tannic acid (TA) was purchased from Shanghai Titan Scientific Co., Ltd (Shanghai, China). CHO‐PEG5k‐CHO was purchased from Tanshtech Co., Ltd (Guangzhou, China). Zn(NO_3_)_2_·6H_2_O was purchased from Yonghua Chemical Co., Ltd (Suzhou, China). Total Antioxidant Capacity Assay Kit with a Rapid ABTS method was purchased from Beyotime Biotech Inc (Shanghai, China). Lysotracker Red DND‐99 was purchased from Yeasen Biotechnology Co., Ltd. (Shanghai, China). MTT was purchased from Solarbio Science & Technology Co., Ltd (Beijing, China). Benzalkonium chloride (BAC) was purchased from Tokyo Chemical Industry Co., Ltd (Tokyo, Japan).

### 4.2 Preparation of Dual‐Dynamically Crosslinked Nanogels

BBR@MPN‑NGs were synthesized via a multi‑step crosslinking strategy. First, a primary network was formed through a Schiff base reaction between the aldehyde groups of PEG‐CHO and the amino groups of CMCS. A 22.3 g L^−1^ solution of PEG‐CHO was dissolved in ethanol, and a 14.875 g L^−1^ solution of CMCS was prepared in water. Separately, 214 µL of the PEG‐CHO solution and 320 µL of the CMCS solution were dissolved in 2 mL of water and incubated overnight at 37°C. Subsequently, a mixed solution of TA and BBR was prepared by adding 160 µL of aqueous TA solution (20 g L^−1^) and 47 µL of BBR solution in DMSO (10 mM) into 5 mL of deionized water, followed by thorough mixing at 500 rpm and room temperature. Then, the pre‐formed CMCS‑PEG network solution was added to the TA/BBR mixture, followed by stirring at 500 rpm and 37°C for 10 min. Finally, 1 mL of an aqueous solution of Zn(NO_3_)_2_·6H_2_O (2 g L^−1^) was added, and stirring was continued at 500 rpm and room temperature for another 10 min. The obtained nanoparticles were collected by centrifugation at 8000 rpm for 15 min and washed twice with deionized water. MPN‑NGs, C6@MPN‑NGs, and NR@MPN‑NGs were prepared analogously using the corresponding drug or dye. For C6@MPN(OCH_3_) and BBR@MPN(OCH_3_), the aldehyde‑modified PEG was replaced by methoxypolyethylene glycol.

### 4.3 In Vitro Antioxidant Activity of BBR@MPN‐NGs in Cells

Intracellular ROS levels were measured using the DCFH‑DA probe. To induce oxidative stress, HCECs were exposed to H_2_O_2_ (350 µm, 1.5 h) or HS (450 mOsm L^−1^, 20 h), while RAW264.7 cells were stimulated with lipopolysaccharide (LPS, 1 µg mL^−^^1^). HCECs (1 × 10^5^ cells per well) and RAW264.7 cells (5 × 10^5^ cells per well) were seeded in 24‐well plates and cultured overnight. HCECs were pretreated with MPN‐NGs, free BBR, or BBR@MPN‐NGs (final concentrations: BBR, 3.5 µM; TA, 11.9 µg mL^−1^) for 2 h before the addition of H_2_O_2_ or HS medium. RAW264.7 cells were pretreated with LPS for 1 h and then cultured in medium containing MPN‐NGs, BBR, and BBR@MPN‐NGs (final concentrations: BBR, 3.5 µm; TA, 11.9 µg mL^−1^) for 20 h. After incubation, cells were washed thoroughly and incubated with 10 µm DCFH‐DA in serum‐free DMEM for 30 min at 37°C in the dark. Subsequently, the cells were washed with PBS for 3 times, and fluorescent images were captured using a fluorescence microscope (IX73, Olympus, Japan). Using a similar protocol, the mean DCF fluorescence intensity was quantified by a flow cytometer (FACSCalibur, BD Biosciences, USA).

### 4.4 In Vitro Anti‐Inflammatory Effect of BBR@MPN‐NGs in Cells

To evaluate the anti‑inflammatory effects of BBR@MPN‑NGs, qPCR was performed. HCECs and RAW264.7 cells were seeded in 12‐well plates at densities of 3 × 10^5^ and 1 × 10^6^ cells per well, respectively, and cultured overnight. HCECs were pretreated with MPN‐NGs, free BBR, or BBR@MPN‐NGs (BBR: 3.5 µm, TA: 11.9 µg mL^−1^ at final concentrations) for 2 h, followed by exposure to HS (450 mOsm L^−1^) for 20 h. To further investigate the functional involvement of JNK/MAPK signaling in the anti‐inflammatory effects of BBR@MPN‐NGs, rescue experiments were performed using anisomycin, a pharmacological activator of JNK/p38 signaling. HCECs were pretreated with BBR@MPN‐NGs in the presence or absence of anisomycin (1 µM) for 2 h, followed by exposure to HS (450 mOsm L^−^^1^) for 20 h. RAW264.7 cells were first stimulated with 1 µg mL^−1^ LPS for 1 h, then the medium was replaced with medium containing BBR, MPN‐NGs, or BBR@MPN‐NGs (BBR: 3.5 µM, TA: 11.9 µg mL^−1^ at final concentrations), and incubation was continued for 20 h. Total RNA was then extracted and subjected to qPCR analysis to determine the expression levels of inflammatory cytokines.

### 4.5 RNA Sequencing and Bioinformatics Analysis

HCECs and RAW264.7 cells were seeded in 6‐well plates at densities of 6 × 10^5^ and 2 × 10^6^ cells per well, respectively, and cultured overnight. HCECs were pretreated with BBR@MPN‐NGs (BBR 3.5 µM, TA 11.9 µg mL^−1^) for 2 h, followed by HS (450 mOsm L^−1^) for 20 h, and RAW264.7 cells were stimulated with LPS (1 µg mL^−^^1^) for 1 h, followed by BBR@MPN‐NGs treatment for 20 h. Total RNA was extracted using TRIzol Reagent (Invitrogen), and RNA quality was assessed with a NanoDrop 2000 spectrophotometer and an Agilent 5300 Bioanalyzer. Only samples with OD260/280 of 1.8‐2.2 and RQN ≥ 6.5 were used for library preparation. Libraries were constructed with the Illumina Stranded mRNA Prep Ligation kit using 1 µg of total RNA and sequenced on an Illumina NovaSeq X Plus platform (2 × 150 bp paired‐end). Raw reads were quality‐controlled with fastp and aligned to the human or mouse reference genome for HCEC and RAW264.7 samples, respectively, using HISAT2. Gene expression was quantified with RSEM and normalized as transcripts per million reads (TPM). Differential expression analysis was performed with DESeq2, with fold change ≥ 1.5 and p ≤ 0.05 as the significance thresholds. GO functional enrichment and KEGG pathway enrichment analyses of differential genes were carried out with the clusterProfiler R software package. The sequencing data have been deposited in GEO (accession number: GSE346535).

### 4.6 BAC‐Induced DED Murine Model

All animal experiments were approved by the Laboratory Animal Research Center of Sir Run Run Shaw Hospital, Zhejiang University School of Medicine. The animal protocols were assessed and approved by the Animal Experimental Ethical Inspection of the Sir Run Run Shaw Hospital, Zhejiang University School of Medicine (grant number SRRSH2025‐0061). Wild‐type C57BL/6 mice (male, 7–8 weeks, 20 ± 2 g) were purchased from Hangzhou Ziyuan Laboratory Animal Technology Co., Ltd. A total of 54 mice were used and housed in a standard environment with a constant temperature (22 ± 1°C) and a regular 12 h light/12 h dark cycle, with free access to standard food and water. Experimental DED in mice was induced using BAC. Briefly, 5 µL of 0.2% BAC in PBS (w/v) was topically administered to the eyes of each mouse twice daily for 7 consecutive days. Subsequently, the 48 successfully modeled DED mice were randomly divided into eight groups (*n* = 6 per group), and a separate normal control group (n = 6) was included. Specifically, four groups received 5 µL of PBS, MPN‐NGs (TA: 681.2 µg mL^−1^), BBR (200 µm), or BBR@MPN‐NGs (BBR: 200 µm, TA: 681.2 µg mL^−1^) topically twice daily for 4 consecutive days, whereas the other four groups received 5 µL of PBS, BBR@MPN‐NGs (BBR: 200 µm, TA: 681.2 µg mL^−^^1^), CsA, or DEX topically once daily for 4 consecutive days. Meanwhile, mice in the normal group received 5 µL of PBS as a control.

### 4.7 Scopolamine‐Induced DED Murine Model

Wild‐type C57BL/6 mice (male, 7–8 weeks, 20 ± 2 g) were used for establishing scopolamine‐induced dry eye models. A 2.5 mg mL^−1^ solution of scopolamine hydrobromide (SH) (MedChemExpress, HY‐N0296A) was prepared in 0.9% saline. In the first week, A total of 36 mice in the DED groups received subcutaneous injections of 0.5 mg/0.2 mL SH solution four times daily for 7 consecutive days. Subsequently, the mice were randomly divided into six groups (*n* = 6) and received topical eye drops of various agents once daily from day 8.

### 4.8 Therapeutic Efficacy Assessment

All mice were anesthetized, and the therapeutic efficacy was evaluated by clinical assessments for dry eye, including ocular surface scores determined by sodium fluorescein staining, tear break‐up time (TBUT), and tear volume measured by the phenol red thread test. For ocular sodium fluorescein staining, 2 µL of 1% sodium fluorescein (w/v) was instilled into the inferior conjunctival sac and eyelid, followed by gentle closing of the eyelid 3–4 times. Fluorescein staining images were captured under cobalt blue light using a slit‐lamp microscope (YZ5E, 66 Vision‐Tech Co., Ltd.), and fluorescein staining scores were graded as follows: each quadrant was scored on a scale of 0–4: 0, no staining; 1, mild punctate staining with < 30 spots; 2, punctate staining with > 30 spots but not diffuse; 3, severe diffuse staining without positive plaques; 4, positive fluorescein plaque staining. Six eyes per group were examined by investigators blinded to group allocation, and the average scores were analyzed. TBUT was measured under cobalt blue light using a slit‐lamp imaging system, with timing stopped at the appearance of the first dark spot and the duration recorded accordingly. Tear volume was measured using phenol red‐impregnated cotton threads (Jing Ming Tech Co., Ltd), which were placed on the lower eyelid palpebral conjunctiva at a site 1/3 from the lateral canthus for 15 s, and the length of the wetted cotton thread was measured.

### 4.9 Statistical Analysis

The data from the pharmacokinetic test of BBR in corneal tissue were presented as mean ± standard error of the mean (SEM), and all other data were presented as mean ± standard deviation (SD). The sample size (n) for each experiment is indicated in the corresponding figure legend. For all datasets with n ≥ 6 per group, normality was assessed for each group using the Shapiro‐Wilk test. Normally distributed data were analyzed by one‐way ANOVA followed by Tukey's multiple comparisons test, while non‐normally distributed data were analyzed using the Kruskal‐Wallis test followed by Dunn's multiple comparisons test. For experiments with *n* = 3 per group, where formal normality testing is statistically underpowered, data were assumed to be normally distributed based on the distributional properties of the respective measurements. Two‐sided tests were used throughout, and the significance thresholds were set as ns *p* > 0.05, ^*^ *p* < 0.05, ^**^ *p* < 0.01, ^***^ *p* < 0.001, and ^****^ *p* < 0.0001. All statistical analyses were performed using GraphPad Prism 8.0 Software.

### 4.10 AI Declaration

No AI tools were used in the preparation of any figures in this manuscript. All schematic diagrams and the Table of Contents image were created by the authors using Adobe Illustrator under an institutional license.

## Author Contributions

**Yanru Xin**: conceptualization, writing – original draft, methodology, data curation, investigation, validation, formal analysis, supervision, project administration, funding acquisition. **Sisi Chen**: methodology, investigation, funding acquisition, formal analysis, data curation. **Shengli Huang**: methodology, investigation, funding acquisition. **Mengqi Huang**: methodology, investigation. **Jingyan Zhu**: methodology, investigation. **Zijian Wang**: methodology, investigation. **Jianliang Shen**: writing – review and editing, supervision, resources, conceptualization. **Qinxiang Zheng**: conceptualization, writing – review and editing, supervision, funding acquisition, resources.

## Conflicts of Interest

The authors declare no conflicts of interest.

## Supporting information




**Supporting File**: advs78237‐sup‐0001‐SuppMat.docx.

## Data Availability

The transcriptomics data have been deposited in the NCBI Gene Expression Omnibus (GEO) under accession number GSE346535. All the other data supporting the findings of this study are available from the corresponding author upon reasonable request.

## References

[1] J. A. Clayton , “Dry Eye,” New England Journal of Medicine 378, no. 23 (2018): 2212–2223, 10.1056/NEJMra1407936. 29874529

[2] F. E. Hakim and A. V. Farooq , “Dry Eye Disease,” Jama 327, no. 5 (2022): 478–479, 10.1001/jama.2021.19963. 35103781

[3] A. J. Bron , C. S. de Paiva , S. K. Chauhan , et al., “TFOS DEWS II Pathophysiology Report,” The Ocular Surface 15, no. 3 (2017): 438–510, 10.1016/j.jtos.2017.05.011. 28736340

[4] M. Farid , “Dry Eye Disease: Let's Start Thinking Outside of the Artificial Tear Box,” Ophthalmology 124, no. 11 (2017): S1–S3, 10.1016/j.ophtha.2017.07.021. 29055356

[5] C. S. de Paiva , S. C. Pflugfelder , S. M. Ng , and E. K. Akpek , “Topical Cyclosporine A Therapy for Dry Eye Syndrome,” (2019), CD010051, 10.1002/14651858.CD010051.pub2. PMC6743670 31517988

[6] S. M. Hoy , “Ciclosporin Ophthalmic Emulsion 0.1%: A Review in Severe Dry Eye Disease,” Drugs 77, no. 17 (2017): 1909–1916, 10.1007/s40265-017-0834-x. 29110189

[7] R. Mazet , J. B. G. Yaméogo , D. Wouessidjewe , L. Choisnard , and A. Gèze , “Recent Advances in the Design of Topical Ophthalmic Delivery Systems in the Treatment of Ocular Surface Inflammation and Their Biopharmaceutical Evaluation,” Pharmaceutics 12, no. 6 (2020): 570, 10.3390/pharmaceutics12060570. PMC7356360 32575411

[8] C. Baudouin , A. Labbé , H. Liang , A. Pauly , and F. Brignole‐Baudouin , “Preservatives in Eyedrops: The Good, the Bad and the Ugly,” Progress in Retinal and Eye Research 29, no. 4 (2010): 312–334, 10.1016/j.preteyeres.2010.03.001. 20302969

[9] R. Langer , “New Methods of Drug Delivery,” Science 249, no. 4976 (1990): 1527–1533, 10.1126/science.2218494. 2218494

[10] Y. C. Kim , M. D. Shin , S. F. Hackett , et al., “Gelling Hypotonic Polymer Solution for Extended Topical Drug Delivery to the Eye,” Nature Biomedical Engineering 4, no. 11 (2020): 1053–1062, 10.1038/s41551-020-00606-8. PMC7655548 32895514

[11] M. Matsuzawa , T. Ando , S. Fukase , et al., “The Protective Role of Conjunctival Goblet Cell Mucin Sialylation,” Nature Communications 14, no. 1 (2023): 1417, 10.1038/s41467-023-37101-y. PMC10023771 36932081

[12] A. Urtti , “Challenges and Obstacles of Ocular Pharmacokinetics and Drug Delivery,” Advanced Drug Delivery Reviews 58, no. 11 (2006): 1131–1135, 10.1016/j.addr.2006.07.027. 17097758

[13] C. Wang , X. Liu , and W. Lv , “Long‐Lasting Comfort Ocular Surface Drug Delivery by in Situ Formation of an Adhesive Lubricative Janus Nanocoating,” Science Advances 11, no. 10 (2025): ads0282, 10.1126/sciadv.ads0282. PMC11887845 40053587

[14] J. Shen , L. Chen , X. Lv , et al., “Emerging Co‐Assembled and Sustained Released Natural Medicinal Nanoparticles for Multitarget Therapy of Choroidal Neovascularization,” Advanced Materials 36, no. 23 (2024): 2314095, 10.1002/adma.202314095. 38344832

[15] Z. Wang , Z. Lv , Y. Ge , et al., “Self‐Polymerized Polyphenol‐Based Platform for the Management of Dry Eye Pathogenesis,” Nature Communications 17, no. 1 (2026): 4132, 10.1038/s41467-026-70388-1. PMC13149814 41851101

[16] Y. Wang , Y. Hu , J. An , et al., “Liposome‐Based Permeable Eyedrops for Effective Posterior Segment Drug Delivery,” Advanced Functional Materials 34, no. 39 (2024): 2403142, 10.1002/adfm.202403142.

[17] L. Ou , Z. Wu , X. Hu , et al., “A Tissue‐Adhesive F127 Hydrogel Delivers Antioxidative Copper‐Selenide Nanoparticles for the Treatment of Dry Eye Disease,” Acta Biomaterialia 175 (2024): 353–368, 10.1016/j.actbio.2023.12.021. 38110136

[18] W. Park , V. P. Nguyen , Y. Jeon , et al., “Biodegradable Silicon Nanoneedles for Ocular Drug Delivery,” Science Advances 8, no. 13 (2022): abn1772, 10.1126/sciadv.abn1772. PMC8967230 35353558

[19] S. Li , L. Chen , and Y. Fu , “Nanotechnology‐Based Ocular Drug Delivery Systems: Recent Advances and Future Prospects,” Journal of Nanobiotechnology 21, no. 1 (2023): 232, 10.1186/s12951-023-01992-2. PMC10362606 37480102

[20] K. Zhang , L. Zhang , and R. N. Weinreb , “Ophthalmic Drug Discovery: Novel Targets and Mechanisms for Retinal Diseases and Glaucoma,” Nature Reviews Drug Discovery 11, no. 7 (2012): 541–559, 10.1038/nrd3745. 22699774

[21] B. Unnikrishnan , J.‐Y. Lai , A. Anand , C.‐C. Huang , and H.‐T. Chang , “Tiny Tools, Big Vision: A Minireview on Carbonized Nanomaterials in Ophthalmology,” Journal of Controlled Release 388 (2025): 114396, 10.1016/j.jconrel.2025.114396. 41232805

[22] S. M. Mousavi , M. Y. Kalashgrani , C.‐J. Yang , and J.‐Y. Lai , “Recent Advances in the Polysaccharide Nanogel Design for Dry Eye Therapy,” Carbohydrate Polymers 382 (2026): 125223, 10.1016/j.carbpol.2026.125223. 42002315

[23] Y. Han , L. Jiang , H. Shi , et al., “Effectiveness of an Ocular Adhesive Polyhedral Oligomeric Silsesquioxane Hybrid Thermo‐Responsive FK506 Hydrogel in a Murine Model of Dry Eye,” Bioactive Materials 9 (2022): 77–91, 10.1016/j.bioactmat.2021.07.027. PMC8586264 34820557

[24] T. Chen , R. Liu , Q. Chen , X. Feng , B. Kong , and Y. Zhao , “MSC‐Encapsulated Porous Microparticle Eye Drops for Autoimmune Dry Eye Disease Treatment in NOD Mice,” Science Advances 11, no. 39 (2025): adu9772, 10.1126/sciadv.adu9772. PMC12459467 40991713

[25] S. Ghosh , C. J. Yang , and J. Y. Lai , “Hollow Nanoarchitectures: Materials Engineering, Nanochemistry, and Biomedical Applications,” Progress in Materials Science 158 (2026): 101634, 10.1016/j.pmatsci.2025.101634.

[26] H. Ejima , J. J. Richardson , K. Liang , et al., “One‐Step Assembly of Coordination Complexes for Versatile Film and Particle Engineering,” Science 341, no. 6142 (2013): 154–157, 10.1126/science.1237265. 23846899

[27] H. Geng , Q.‐Z. Zhong , J. Li , et al., “Metal Ion‐Directed Functional Metal–Phenolic Materials,” Chemical Reviews 122, no. 13 (2022): 11432–11473, 10.1021/acs.chemrev.1c01042. 35537069

[28] Z. Lin , H. Liu , J. J. Richardson , et al., “Metal–phenolic network composites: From fundamentals to applications,” Chemical Society Reviews 53, no. 22 (2024): 10800–10826, 10.1039/d3cs00273j. 39364569

[29] J. Chen , S. Pan , J. Zhou , et al., “Assembly of Bioactive Nanoparticles via Metal–Phenolic Complexation,” Advanced Materials 34, no. 10 (2022): 2108624, 10.1002/adma.202108624. 34933398

[30] R. Yu , H. Chen , J. He , et al., “Engineering Antimicrobial Metal–Phenolic Network Nanoparticles With High Biocompatibility for Wound Healing,” Advanced Materials 36, no. 6 (2024): 2307680, 10.1002/adma.202307680. 37997498

[31] W. Xu , Z. Lin , S. Pan , et al., “Direct Assembly of Metal‐Phenolic Network Nanoparticles for Biomedical Applications,” Angewandte Chemie International Edition 62, no. 45 (2023): 202312925, 10.1002/anie.202312925. PMC10953434 37800651

[32] Z. Wei , G. Peng , Y. Zhao , et al., “Engineering Antioxidative Cascade Metal‐Phenolic Nanozymes for Alleviating Oxidative Stress During Extracorporeal Blood Purification,” ACS Nano 16, no. 11 (2022): 18329–18343, 10.1021/acsnano.2c06186. 36356207

[33] G. Liu , K. Li , H. Wang , L. Ma , L. Yu , and Y. Nie , “Stable Fabrication of Zwitterionic Coating Based on Copper‐Phenolic Networks on Contact Lens With Improved Surface Wettability and Broad‐Spectrum Antimicrobial Activity,” ACS Applied Materials & Interfaces 12, no. 14 (2020): 16125–16136, 10.1021/acsami.0c02143. 32202402

[34] A. Mishra , J. Halder , R. K. Sahoo , et al., “Hybrid Nanogel System of Quercetin‐Loaded Squalene NLCs With Zinc–HA Network for Dry Eye Syndrome,” International Journal of Biological Macromolecules 353 (2026): 151215, 10.1016/j.ijbiomac.2026.151215. 41802516

[35] Z. Lin , J. J. Richardson , J. Zhou , and F. Caruso , “Direct Synthesis of Amorphous Coordination Polymers and Metal–Organic Frameworks,” Nature Reviews Chemistry 7, no. 4 (2023): 273–286, 10.1038/s41570-023-00474-1. 37117419

[36] Q.‐Z. Zhong , S. Li , J. Chen , et al., “Oxidation‐Mediated Kinetic Strategies for Engineering Metal–Phenolic Networks,” Angewandte Chemie International Edition 58, no. 36 (2019): 12563–12568, 10.1002/anie.201907666. 31318144

[37] Z. J. Jia , M. Wen , Y. Cheng , and Y. F. Zheng , “Strategic Advances in Spatiotemporal Control of Bioinspired Phenolic Chemistries in Materials Science,” Advanced Functional Materials 31, no. 14 (2021): 2008821, 10.1002/adfm.202008821.

[38] C. Chen , M. Lin , C. Wahl , et al., “Dynamic Metal–Phenolic Coordination Complexes for Versatile Surface Nanopatterning,” Journal of the American Chemical Society 145, no. 14 (2023): 7974–7982, 10.1021/jacs.2c13515. 36975188

[39] L. Zhu , T. Yu , W. Wang , et al., “Responsively Degradable Nanoarmor‐Assisted Super Resistance and Stable Colonization of Probiotics for Enhanced Inflammation‐Targeted Delivery,” Advanced Materials 36, no. 18 (2024): 2308728, 10.1002/adma.202308728. 38241751

[40] N. Holten‐Andersen , M. J. Harrington , H. Birkedal , et al., “pH‐Induced Metal‐Ligand Cross‐Links Inspired by Mussel Yield Self‐Healing Polymer Networks With Near‐Covalent Elastic Moduli,” Proceedings of the National Academy of Sciences 108, no. 7 (2011): 2651–2655, 10.1073/pnas.1015862108. PMC3041094 21278337

[41] J.‐Y. Sun , X. Zhao , W. R. K. Illeperuma , et al., “Highly Stretchable and Tough Hydrogels,” Nature 489, no. 7414 (2012): 133–136, 10.1038/nature11409. PMC3642868 22955625

[42] X. Li and J. P. Gong , “Design Principles for Strong and Tough Hydrogels,” Nature Reviews Materials 9, no. 6 (2024): 380–398, 10.1038/s41578-024-00672-3.

[43] D. Li , J. Li , S. Wang , Q. Wang , and W. Teng , “Dually Crosslinked Copper‐Poly(tannic acid) Nanoparticles With Microenvironment‐Responsiveness for Infected Wound Treatment,” Advanced Healthcare Materials 12, no. 17 (2023): 2203063, 10.1002/adhm.202203063. 36842067

[44] M. A. Aboudzadeh , A. Iturrospe , A. Arbe , M. Grzelczak , and F. Barroso‐Bujans , “Cyclic Polyethylene Glycol as Nanoparticle Surface Ligand,” ACS Macro Letters 9, no. 11 (2020): 1604–1610, 10.1021/acsmacrolett.0c00730. 35617061

[45] M. Shin , J. H. Ryu , J. P. Park , et al., “DNA/Tannic Acid Hybrid Gel Exhibiting Biodegradability, Extensibility, Tissue Adhesiveness, and Hemostatic Ability,” Advanced Functional Materials 25, no. 8 (2015): 1270–1278, 10.1002/adfm.201403992.

[46] Y. Han , S. Guo , Y. Li , et al., “Berberine Ameliorate Inflammation and Apoptosis via Modulating PI3K/AKT/NFκB and MAPK Pathway on Dry Eye,” Phytomedicine 121 (2023): 155081, 10.1016/j.phymed.2023.155081. 37748390

[47] N. Chaiwong , Y. Phimolsiripol , P. Leelapornpisid , et al., “Synergistics of Carboxymethyl Chitosan and Mangosteen Extract as Enhancing Moisturizing, Antioxidant, Antibacterial, and Deodorizing Properties in Emulsion Cream,” Polymers 14, no. 1 (2022): 178, 10.3390/polym14010178. PMC8747190 35012200

[48] S. Pourmohammadi , P. Valipour , M. Hosseinzadeh , R. Khajavi , and M. A. Khalilzadeh , “Bioactive Sago Starch Films Incorporating Tannic Acid and Nanocellulose: Improved Antimicrobial and Barrier Performance for Prolonged Shelf Life,” International Journal of Biological Macromolecules 366 (2026): 150859, 10.1016/j.ijbiomac.2026.150859. 41780777

[49] Y. Wu , Y. Wang , L. Long , C. Hu , Q. Kong , and Y. Wang , “A Spatiotemporal Release Platform Based on pH/ROS Stimuli‐Responsive Hydrogel in Wound Repairing,” Journal of Controlled Release 341 (2022): 147–165, 10.1016/j.jconrel.2021.11.027. 34813880

[50] A. Khojastehnezhad , A. Bisio , S. F. Rafie , et al., “Hierarchical Design of COFs via NHC‐Grafting for Selective Gold Recovery From E‐Waste: Experimental and MD Analyses,” Journal of the American Chemical Society 147, no. 42 (2025): 38516–38533, 10.1021/jacs.5c12234. 41071603

[51] F. Ding , Y. Zhang , X. Huang , J. Shi , Y. Li , and X. Zou , “Carboxymethyl Chitin Nanofibers Stabilized Pickering Emulsion Gel Crosslinked by Oxidized Alginate for Improving Low‐Fat Pork Gel Quality,” International Journal of Biological Macromolecules 344 (2026): 150206, 10.1016/j.ijbiomac.2026.150206. 41539522

[52] W. Hong , H. Xiao , Z. Zhang , et al., “A Composite Film of Oxidized Levan Crosslinked With Gelatin: Strong Hydrophobic and Mechanical Properties and Effective Preservation of Fresh Mozzarella Cheese,” International Journal of Biological Macromolecules 284 (2025): 137701, 10.1016/j.ijbiomac.2024.137701. 39549806

[53] T. Lan , Y. Dong , J. Shi , et al., “Advancing Self‐Healing Soy Protein Hydrogel With Dynamic Schiff Base and Metal‐Ligand Bonds for Diabetic Chronic Wound Recovery,” Aggregate 5, no. 6 (2024): 639, 10.1002/agt2.639.

[54] M. Romio , L. Trachsel , G. Morgese , S. N. Ramakrishna , N. D. Spencer , and E. M. Benetti , “Topological Polymer Chemistry Enters Materials Science: Expanding the Applicability of Cyclic Polymers,” ACS Macro Letters 9, no. 7 (2020): 1024–1033, 10.1021/acsmacrolett.0c00358. 35648599

[55] H. L. Bui , Y. H. Su , C. J. Yang , C. J. Huang , and J. Y. Lai , “Mucoadhesive, Antioxidant, and Lubricant Catechol‐Functionalized Poly(phosphobetaine) as Biomaterial Nanotherapeutics for Treating Ocular Dryness,” Journal of Nanobiotechnology 22, no. 1 (2024): 160, 10.1186/s12951-024-02448-x. PMC11000383 38589911

[56] H. Liu , C. Begley , M. Chen , et al., “A Link between Tear Instability and Hyperosmolarity in Dry Eye,” Investigative Opthalmology & Visual Science 50, no. 8 (2009): 3671–3679, 10.1167/iovs.08-2689. 19324847

[57] S. Li , Z. Lu , Y. Huang , et al., “Anti‐Oxidative and Anti‐Inflammatory Micelles: Break the Dry Eye Vicious Cycle,” Advanced Science 9, no. 17 (2022): 2200435, 10.1002/advs.202200435. PMC9189644 35435328

[58] J. G. Galletti , M. Guzmán , and M. N. Giordano , “Mucosal Immune Tolerance at the Ocular Surface in Health and Disease,” Immunology 150, no. 4 (2017): 397–407, 10.1111/imm.12716. PMC5343370 28108991

[59] Z. Liu , H. E. Xie , L. Li , et al., “Single‐Cell Landscape Reveals the Epithelial Cell‐Centric Pro‐Inflammatory Immune Microenvironment in Dry Eye Development,” Mucosal Immunology 17, no. 3 (2024): 491–507, 10.1016/j.mucimm.2023.11.008. 38007004

[60] E. M. del Amo , “Topical Ophthalmic Administration: Can a Drug Instilled Onto the Ocular Surface Exert an Effect at the Back of the Eye?,” Frontiers in Drug Delivery 2 (2022): 954771, 10.3389/fddev.2022.954771.

[61] A. Loiseau , G. Raîche‐Marcoux , C. Maranda , N. Bertrand , and E. Boisselier , “Animal Models in Eye Research: Focus on Corneal Pathologies,” International Journal of Molecular Sciences 24, no. 23 (2023): 16661, 10.3390/ijms242316661. PMC10706114 38068983

