# Supplementary material for: Dual‐Dynamically Crosslinked Nanogels Enable Prolonged Ocular Retention for Multimodal Dry Eye Therapy
Source: Adv Sci (Weinh). 2026 Oct 11:e78237. Online ahead of print. doi: 10.1002/advs.78237 (PMC13655254; doi:10.1002/advs.78237)
Supplement: Supplementary file 1 [file ADVS-9999-e78237-s001.docx]

Supporting Information

Dual-Dynamically Crosslinked Nanogels Enable Prolonged Ocular Retention for Multimodal Dry Eye Therapy

Yanru Xin^1†^*, Sisi Chen^1†^, Shengli Huang^1^, Mengqi Huang^1^, Jingyan Zhu^4^, Zijian Wang^4^, Jianliang Shen^2,3^*, Qinxiang Zheng^1^*

^1^ Department of Ophthalmology, Sir Run Run Shaw Hospital, Zhejiang University School of Medicine, Hangzhou 310016, P. R. China.

^2^ Zhejiang Key Laboratory of Ophthalmic Drug Discovery and Medical Device Research, Eye Hospital, Wenzhou Medical University, Wenzhou 325027, P. R. China

^3^ Zhejiang Engineering Research Center for Tissue Repair Materials, Wenzhou Institute, University of Chinese Academy of Sciences, Wenzhou 325001, P. R. China.

^4^ National Clinical Research Center for Ocular Diseases, Eye Hospital, Wenzhou Medical University, Wenzhou 325000, P. R. China.

^†^ These authors contributed equally as co-first authors.

* [yanruxin@zju.edu.cn](mailto:yanruxin@zju.edu.cn) (Y. Xin); shenjl@wiucas.ac.cn or sjl1@wmu.edu.cn (J. Shen); qinxiangzheng@zju.edu.cn (Q. Zheng).

**Methods**

**Characterizations of BBR@MPN-NGs.**

The morphology and surface element composition of MPN-NGs and BBR@MPN-NGs were examined by transmission electron microscopy (TEM, Hitachi HT7820, Japan). Hydrodynamic diameter and zeta potential were measured by dynamic light scattering (DLS) using a Malvern Zetasizer Pro. UV-vis absorption spectra were recorded on a multifunctional microplate reader (SpectraMax iD5, Molecular Devices, USA) in quartz microplates. Fourier-transform infrared (FTIR) spectra were acquired at room temperature on a Nicolet iS50 spectrometer (Thermo Scientific, USA). X-ray photoelectron spectroscopy (XPS) were performed on a Kratos Axis Supra^+^ spectrometer and data were analyzed with CasaXPS software. ^1^H NMR spectra were recorded on an NMR spectrometer (AVANCEIII 500MHz, Bruker). DMSO‑d₆ was used as the solvent for NMR measurement. The concentration of BBR was calculated based on the characteristic peak at 421 nm. ICP-MS (Agilent 8900, Agilent Technologies, USA) and the Folin phenol assay were used to determine the Zn²⁺ and TA loading efficiency, respectively. Loading efficiency (LE) and encapsulation efficiency (EE) were calculated as follows:

LE (%) = (W_1_/W_0_) × 100%

EE (%) = (W_1_/W_2_) × 100%

Where W_1_ is the weight of loaded BBR, W_0_ is the total amount of nanoparticles, and W_2_ is the weight of BBR initially added.

During storage, the TA content was determined by the Folin-Ciocalteu method. BBR retention was assessed by ultrafiltration. After storage, free BBR was separated from the nanogels and quantified in the filtrate. BBR retention was calculated as follows:

BBR retention (%) = (M_0_-M_free, t_)/M_0_ × 100%

Where M_0_ is the initial BBR amount in the purified formulation before storage, and M_free, t_ is the amount of free BBR in the corresponding sample at storage time t, determined from the ultrafiltrate.

***In vitro* release of BBR from BBR@MPN-NGs.**

The release rate of BBR from BBR@MPN-NGs was evaluated using the dialysis method. A 1 mL aliquot of BBR@MPN-NGs dispersion was sealed in a dialysis bag (MWCO 7000) and immersed in 10 mL of simulated tear fluid (Mimic FluiX, MSB02063) supplemented with 1% SDS to maintain sink conditions, at 37 °C under continuous shaking at 200 rpm. The simulated tear fluid was formulated with the principal tear components, including electrolytes, urea, albumin, ascorbic acid, and lysozyme. At predetermined time intervals, 1 mL of the release medium was collected and replaced with an equal volume of fresh release medium. The amount of released BBR was determined by UV-vis absorption at 421 nm.

**Free radical scavenging activity of BBR@MPN-NGs.**

In the DPPH scavenging assay, a fresh DPPH stock solution was prepared by dissolving 0.002 g of DPPH in 50 mL of ethanol. This solution was mixed with an equal volume of BBR@MPN-NGs at various concentrations (1, 2.5, 5, and 10 μg mL^-1^, based on TA concentration). The resulting mixtures were transferred to a 96‑well plate and incubated in the dark for 30 min. The absorbance at 517 nm was then measured using a microplate reader (SpectraMax iD5, Molecular Devices, USA).

For the ABTS radical scavenging assay, the ABTS working solution was prepared according to the manufacturer’s instructions (Beyotime, S0121). Subsequently, 10 μL of BBR@MPN-NGs at various concentrations (1, 2.5, 5, and 10 µg mL^-1^, based on TA concentration) was mixed with 170 μL of the ABTS working solution. The mixture was transferred into a 96-well plate and incubated in the dark for 6 min. The absorbance at 414 nm was then measured using a microplate reader. The scavenging efficiency was calculated using the following formula:

$$\text{S}\text{cavenging effect }\left( \text{\%} \right)\text{=1-}\frac{\text{A}_{\text{e}}\text{-}\text{A}_{\text{s}}}{\text{A}_{\text{b}}}\text{×1}\text{00\%}$$

Where A_e_ is the experimental group, A_s_ is the sample group, and A_b_ is the blank group.

**Friction measurements**

To evaluate the lubricating properties of the BBR@MPN-NGs, friction tests were performed on bare silicon wafers and on wafers coated with either commercial polyethylene glycol eye drops (PGEDs; SiRan, Alcon ) or the BBR@MPN-NGs. To prepare the eye drop- or BBR@MPN-NG-coated samples, bare silicon wafers were directly immersed in the PGEDs and BBR@MPN-NGs (1 mg mL^-1^, based on TA concentration) solution at room temperature for 1 h. After coating, the wafers were placed in an oven and dried at 30 °C for 45 min before the friction measurements. The coefficient of friction (COF) was measured using a multifunctional tribometer (MFT-5000, Rtec, USA). In brief, a silicon wafer (either surface-modified or unmodified) was fixed on the lower specimen holder. An aluminum ball was used as the upper half of the friction couple. A normal load of 0.5 N was applied, and the wafer was moved reciprocally against the aluminum ball at a sliding speed of 2.5 mm s^-^¹ over a stroke length of 130 mm. Three sliding cycles were performed for each test to assess the stability of the coating.

**Adhesion of BBR@MPN-NGs to the corneal epithelial cell surface**

To evaluate the adhesion properties of BBR@MPN-NGs, the content of C6@MPN-NGs on the cell surface was determined by C6 fluorescence. C6-loaded liposomes (C6@liposomes) and Schiff base-deficient C6@MPN(OCH₃) served as controls. HCECs were seeded in 24-well plates at 1 × 10⁵ cells per well and cultured for 24 h. Subsequently, C6@MPN-NGs, C6@MPN(OCH₃), or C6@liposomes (all at a C6 concentration of 0.2 µg mL^-1^) were added and incubated with the cells for 1 min. After three PBS washes, the cells were imaged by a fluorescence microscope (IX73, Olympus, Japan), and the intracellular C6 fluorescence was quantified by flow cytometry (CytoFLEX S, Beckman Coulter, USA).

Rapid corneal adhesion was evaluated by measuring Nile red fluorescence of NR@MPN-NGs on excised mouse corneas. Freshly isolated corneas were immersed in NR@MPN-NGs solutions (Nile red, 15 µg mL^-1^; TA, 700 µg mL^-1^) for 2, 5, 10, or 20 s, rinsed three times with PBS, cut into a four‑leaf clover shape, and flattened on glass slides. Confocal imaging was performed on an Olympus FV3000 confocal microscope with excitation at 561 nm and emission collected at 570-659 nm.

**Precorneal retention evaluation**

Precorneal retention was evaluated by *in vivo* fluorescence imaging under anesthesia and by corneal flat-mount analysis in non‑anesthetized mice. For *in vivo* imaging, C57BL/6 mice (aged 7-8 weeks) were anesthetized, and 5 µL of C6@MPN-NGs (C6, 2.22 µg mL^-1^) or free C6 (2.22 µg mL^-1^) was topically administered onto the ocular surface. Fluorescence images were acquired at 0.5, 5, 10, 15, 25, and 35 min post-instillation using the Caliper IVIS Lumina Series III system with excitation at 465 nm and emission at 545 nm, and quantified with IVIS Lumina Living Image Software.

For corneal flat-mount analysis in non-anesthetized mice, 5 μL of NR@MPN-NGs (Nile red, 15 µg mL^-1^; TA, 700 µg mL^-1^) was administered per eye. The mice were sacrificed at 0.5, 1, 2, 4, 8, 12, and 24 h post-administration, respectively, and the corneas were then carefully excised, cut into a four-leaf clover shape, mounted onto glass slides, and immediately imaged by a confocal microscope (FV3000, Olympus, Japan). Nile red fluorescence was semi-quantified using ImageJ software.

**Ocular pharmacokinetics**

Healthy C57BL/6 mice received a single topical instillation of 5 µL of BBR@MPN-NGs or free BBR (200 μM BBR) per eye. At predetermined time points, mice were euthanized and samples were collected. For corneal pharmacokinetics, corneas were excised and corneas from three mice were pooled for each measurement given the limited mass of a single mouse cornea. Pooled corneas were weighed, homogenized in 0.5 mL of pre-cooled methanol/water (1/1, v/v), and sonicated in an ice bath for 30 min. After centrifugation, the supernatants were collected for LC-MS/MS analysis. For tear fluid pharmacokinetics, tear washings were collected following established methods [1, 2] by instilling 3 µL of PBS onto the ocular surface and recovering the fluid, and washings from ten mice per group were pooled into a single sample at each time point. BBR concentrations in corneal homogenates and tear washings were quantified by LC-MS/MS (Shimadzu Nexera X2 LC-30AD coupled to an AB SCIEX 5500 QTRAP).

**Evaluation of cellular internalization profiles of the nanogels**

Cellular uptake of the nanogels was investigated by fluorescence microscopy and flow cytometry. HCECs and RAW264.7 cells were seeded in confocal dishes at densities of 1 × 10⁵ and 3 × 10⁵ cells per well, respectively, and cultured overnight. After incubation with C6@MPN-NGs (C6 concentration, 0.2 µg mL^-1^) for 0.25, 0.5, 1, or 2 h, the cells were washed three times with PBS. Subsequently, the cells were co-incubated with Lyso-Tracker Red DND-99 (50 nM) for 30 min and observed using a confocal laser scanning microscope (FV3000, Olympus, Japan). The excitation wavelength of C6 was 488 nm with signal collection at 500-540 nm, while Lyso-Tracker Red DND-99 was excited at 561 nm and detected at 581-631 nm. For flow cytometry, cells were directly seeded in 24-well plates. After incubation with C6@MPN-NGs (C6 concentration, 0.2 µg mL^-1^) for 0.25, 0.5, 1, and 2 h, cells were digested and harvested with trypsin, washed three times with PBS, and then quantitatively analyzed using a flow cytometer (CytoFLEX S, Beckman Coulter, USA) to determine the mean C6 fluorescence intensity.

***In vitro* anti-apoptotic activity of BBR@MPN-NGs in HCECs**

To assess cytoprotective effects, HCECs were seeded in 96‑well plates at 1 × 10⁴ cells per well and cultured overnight. Cells were preincubated with MPN‑NGs, free BBR, or BBR@MPN‑NGs (BBR: 3.5 µM, TA: 11.9 µg mL^-1^ at final concentrations) for 2 h, followed by exposure to H_2_O_2_ (350 µM) for 3 h. After treatment, cell viability was evaluated by Live/Dead staining using a Calcein/PI Cell Viability and Cytotoxicity Assay Kit according to the manufacturer’s instructions, and fluorescence images were acquired with a microscope (IX73, Olympus, Japan). In parallel, an MTT assay was conducted by incubating identically treated cells with MTT solution (1 mg mL^-1^) for 3.5 h. After removing the MTT, 100 μL of DMSO was added to dissolve the formazan salt. The absorbance at 570 nm was measured using a microplate reader.

***In vitro* macrophage polarization analysis in RAW264.7 cells**

Macrophage polarization was induced by LPS, and CD86 was selected as the marker for the M1 phenotype. FITC anti-mouse CD86 antibody (105005, Biolegend) was used to evaluate M1 polarization of macrophages. RAW264.7 cells were seeded in 24‑well plates at a density of 5 × 10^5^ cells per well and cultured overnight to facilitate cell adhesion. The cells were then stimulated with LPS (1 µg mL^-1^) for 1 h, followed by the addition of BBR@MPN‑NGs (BBR: 3.5 µM, TA: 11.9 µg mL^-1^ at final concentrations) and incubation for 20 h. After collection, cells were processed according to the manufacturer’s instructions and further analyzed by flow cytometer (FACSCanto II, BD Biosciences).

**Western blot analysis**

HCECs were seeded in 12-well plates at densities of 3 × 10⁵ cells per well and cultured overnight. HCECs were pretreated with MPN-NGs, free BBR, or BBR@MPN-NGs (BBR: 3.5 µM, TA: 11.9 µg mL^-1^ at final concentrations) for 2 h, followed by exposure to HS (450 mOsm L^-1^) for 2 h. HCECs were harvested and then lysed with RIPA lysis buffer. After centrifugation at 10,000 rpm for 10 min at 4 °C, the supernatant was collected for Western blot analysis. Protein concentration was determined using the BCA assay kit. Equal amounts of total protein (20 μg) were separated by 10% SDS-PAGE and transferred onto PVDF membranes at 250 mA for 1.5 h. Membranes were blocked with 5% BSA for 1 h and then incubated with the anti-phospho-SAPK/JNK (Thr183/Tyr185) (1:1000 dilution, 4668, Cell Signaling Technology) overnight at 4°C. After washing the membrane with TBST, HRP-conjugated secondary antibody (1:2000 dilution, ZB-2301, ZSGB-Bio) was added and incubated for 1 h at room temperature. Signals were visualized using an enhanced chemiluminescence (ECL) substrate. The membranes were then incubated in stripping buffer (CW0056M, CWBIO) for 1 h to remove bound antibodies, re-blocked, and sequentially re-probed with anti-SAPK/JNK (1:1000 dilution, 9252, Cell Signaling Technology). After a second stripping step, the membranes were re-probed with HRP-conjugated anti-GAPDH (1:10,000 dilution, HRP-60004, Proteintech). All signals for each sample were thus obtained from the same membrane. Band intensities were quantified using ImageJ software. For each sample, p-JNK was normalized to the corresponding total JNK of the same sample. Three independent biological replicates were performed.

**Histological assessment**

After 4 days of treatment, mice in each group were sacrificed according to standard experimental procedures, and their eyeballs and conjunctivae were harvested. Subsequently, hematoxylin and eosin (H&E) staining and periodic acid-Schiff (PAS) staining were performed on these tissues to observe tissue structure. For the detection of ROS, frozen sections were prepared and stained with dihydroethidium (DHE), followed by observation and imaging of DHE fluorescence using a confocal laser scanning microscope (FV3000, Olympus, Japan). The excitation wavelength of DHE was 514 nm, with signal collection at 582-632 nm.

**Immunofluorescence staining**

Sections of the cornea from differently treated mice were fixed, blocked, and incubated overnight at 4 °C with primary antibodies, including anti-IL-1β (1:600, GB11113, Servicebio), anti-TNF-α (1:500, GB115702, Servicebio), and anti-MMP-9 (1:500, GB15132, Servicebio). The sections were then incubated with Cyanine 3-conjugated secondary antibody for 1 h and mounted with DAPI. Afterward, the slides were imaged under a confocal laser scanning microscope (FV3000, Olympus, Japan) and semi-quantitatively analyzed using ImageJ software.

**Macrophages and M1-polarized macrophages percentage assay by flow cytometry**

The percentages of macrophages and M1-polarized macrophages in the cornea, conjunctiva, and lymph nodes were quantitatively determined by flow cytometry. Corneal and conjunctival tissues were carefully dissected and cut into small pieces of approximately 1 mm³. These tissue fragments were incubated in RPMI‑1640 medium containing type Ⅳ collagenase (1 mg mL^-1^) and DNase Ⅰ (0.1 mg mL^-1^) for 1 h in a 37 ℃ water bath shaker for sufficient tissue dissociation. Lymph node tissues were gently mechanically dissociated using a tissue grinder and then subjected to the same enzymatic digestion. The resulting cell suspensions were filtered through a 70 μm nylon strainer to remove undigested tissue fragments. The filtered cells were centrifuged at 300 g for 5 min at 4 ℃, washed with PBS, and resuspended in FACS buffer (PBS supplemented with 2% FBS). The resulting single‑cell suspensions were used for subsequent cell staining and flow cytometric analysis. PE anti‑mouse F4/80 antibody (123110, BioLegend), FITC anti‑mouse CD86 antibody (105005, BioLegend), and Brilliant Violet™ 650 anti‑mouse CD11b antibody (416‑0112‑80, Invitrogen) were added to the cell suspension and incubated for 30 min. Subsequently, 350 μL of FACS buffer was added, gently mixed, and centrifuged at 3000 rpm for 5 min, after which the supernatant was discarded. This washing step was repeated twice to remove the free fluorescent antibodies. The cell precipitate was resuspended in 100 μL of FACS buffer. The cell suspension was transferred to a flow cytometry tube and analyzed using a flow cytometer (CytoFLEX S, Beckman Coulter, USA).

**Sterility** **and bacterial endotoxin tests**

The sterility of the BBR@MPN-NGs formulation was assessed by direct inoculation following the principles of USP <71>. Aliquots of the formulation (0.5 mL) were aseptically inoculated into fluid thioglycollate medium (FTM) and tryptic soy broth (TSB), respectively. The FTM was incubated at 30-35 °C and the TSB at 20-25 °C for 14 days. Media inoculated with Staphylococcus aureus served as positive controls. The formulation was considered sterile when no microbial growth was observed after the incubation period. Bacterial endotoxin levels in the BBR@MPN-NGs formulation were determined using the Limulus Amebocyte Lysate (LAL) gel-clot method (GenScript, L01058) following the principles of USP <85>.

**Zn accumulation assay**

To assess potential Zn accumulation, the wild-type C57BL/6 mice received topical eye instillation once daily with 5 μL BBR@MPN-NGs (BBR: 200 µM, TA: 681.2 µg mL^-^¹) for 1 month. At 0, 14, and 28 days of administration, the cornea and conjunctiva were collected. Given the limited mass and zinc content of individual mouse ocular surface tissues, the cornea and conjunctiva from six mice per group were pooled for each measurement. Pooled tissues were weighed, acid-digested in nitric acid, and Zn concentrations were quantified by ICP-MS (Agilent 7850).

**Safety evaluation**

During 4 consecutive days of treatment in DED mice with 5 μL PBS, BBR (200 μM), MPN-NGs (TA: 681.2 µg mL^-1^), and BBR@MPN-NGs (BBR: 200 µM, TA: 681.2 µg mL^-1^), respectively, body weight was measured at a fixed time (9:00 a.m.) daily. In addition, wild-type C57BL/6 mice were randomly divided into two groups, and each group received topical eye instillation twice daily with 5 μL PBS or BBR@MPN-NGs (BBR: 200 µM, TA: 681.2 µg mL^-1^) for 1 month, respectively. At 0, 7, 14, 21, and 28 days of administration, mouse eyes were examined under a slit-lamp microscope and stained with sodium fluorescein under cobalt blue light. After one month, mice were euthanized, and eyeballs with intact eyelids and conjunctival tissues were carefully enucleated, together with lacrimal glands. H&E staining was performed to evaluate corneal and lacrimal gland morphology, while PAS staining was used to assess conjunctival goblet cells. Major organs, including the heart, liver, spleen, lung, and kidney, were also harvested and prepared for H&E staining to further investigate in vivo biosafety.

**Figure S1.** Zeta potential of BBR@MPN-NGs at various molar ratios of TA to BBR.

**Figure S2.** Stability assay of BBR@MPN(OCH_3_) in the presence of NaCl, Urea, Tween 20, or SDS. Data are mean ± SD (n = 3).

**Figure S3.** FTIR spectra of BBR@MPN-NGs, TA, PEG-CHO, and CMCS at wavenumbers from 800 to 1400 cm^-1^.

**Figure S4.** FTIR spectra of BBR@MPN (OCH_3_) and BBR@MPN-NGs at wavenumbers from 1400 to 1700 cm^-1^.

**Figure S5.** Standard curves of BBR in DMSO.

**Figure S6.** (A) The size of BBR@MPN-NGs in different dispersants in the absence of 0.1% Tween 80. (B) The size of BBR@MPN-NGs in different dispersants in the presence of 0.1% Tween 80. (C) The stability of BBR@MPN-NGs in different dispersants in the presence of 0.1% Tween 80. Data are presented as mean ± SD (n = 3).

**Figure S7.** The stability of (A) MPN-NGs and (B) BBR@MPN-NGs in water at 4 ℃ for 1 year.

**Figure S8.** Long-term stability of BBR@MPN-NGs stored as a concentrated aqueous dispersion at 4 °C protected from light for up to 12 months. (A) Representative TEM images at the indicated time points, showing retained spherical morphology. Scale bar: 500 nm. (B) TA content determined by the Folin phenol method is expressed as a percentage of its initial value at time zero. (C) BBR retention during storage, estimated by ultrafiltration and expressed as a percentage of the initial BBR amount in the purified formulation. (D) DPPH and (E) ABTS radical scavenging efficiencies. Inserts: photos of solutions after different treatments. (F) pH of the dispersion. Data are presented as mean ± SD (n = 3).

**Figure S9.** The size of C6@MPN-NGs, C6@MPN(OCH_3_), and C6@liposomes.

**Figure S10.** The size of NR@MPN-NGs and NR@MPN(OCH_3_).

**Figure S11.** Rapid Adhesion of MPN-NGs to Corneal Tissue. (A) Confocal images and (B) semiquantitative analysis of Nile red fluorescence of corneal flat-mount specimens incubated in NR@MPN-NGs solution for varying durations, then washed three times with PBS before acquisition (n = 3). Scale bar: 500 µm.

**Figure S12.** Pharmacokinetic profiles of BBR in (A) corneal tissue (data presented as mean ± SEM; n = 3 independent pools, each pooling corneas from 3 mice) and (B) tear fluid (data from single pooled samples of tear wash fluids from 10 mice per time point) after a single topical instillation of BBR@MPN-NGs or free BBR, quantified by LC-MS/MS.

**Figure S13.** Contribution of individual nanogel components to anti-inflammatory and antioxidant activity in hyperosmolarity-stimulated HCECs. Relative mRNA expression of (A) TNF-α and (B) IL-23, and (C) intracellular ROS levels quantified by DCF fluorescence intensity, with (D) representative DCFH-DA fluorescence images. Scale bar: 50 μm. Data are presented as mean ± SD (n = 3). Statistical significance was determined using one-way ANOVA (**p* < 0.05, ***p* < 0.01, ****p* < 0.001, *****p* < 0.0001).

**Figure S14.** Protective effects of BBR@MPN-NGs prepared at TA:BBR feeding ratios of 1:0.25 and 1:0.5 against H_2_O_2_-induced cytotoxicity in HCECs, compared at matched BBR concentrations of (A) 2.5, (B) 3.5, and (C) 5 μM. Data are presented as mean ± SD (n = 3). Statistical significance was determined using one-way ANOVA (ns, *p* > 0.05, **p* < 0.05, ***p* < 0.01, ****p* < 0.001, *****p* < 0.0001).

**Figure S15.** Quantitative analysis of p-JNK/MAPK with total JNK/MAPK as a loading control. Data are presented as the mean ± SD (n = 3). Statistical significance was determined using one-way ANOVA (** *p* < 0.01, **** *p* < 0.0001).

**Figure S16.** Effect of JNK/p38 reactivation by anisomycin on the anti-inflammatory efficacy of BBR@MPN-NGs in hyperosmolarity-stimulated HCECs. Relative mRNA expression of (A) TNF-α and (B) IL-23. Data are presented as mean ± SD (n = 3). Statistical significance was determined using one-way ANOVA (ns, *p* > 0.05, **p* < 0.05, ***p* < 0.01, ****p* < 0.001, *****p* < 0.0001).

**Figure S17.** RNA-seq analysis of RAW264.7 cells after BBR@MPN-NGs treatment. (A) Volcano plot showing differentially expressed genes between the LPS and control groups. (B) Venn diagram showing the intersection of significantly upregulated genes in the LPS group and significantly downregulated genes in the BBR@MPN‑NGs group. (C) Volcano plot comparing the LPS + BBR@MPN-NGs and LPS groups.

**Figure S18.** Heat maps of significantly up-regulated genes in the RAW264.7 cells after LPS treatment and down-regulated genes after BBR@MPN-NGs treatment (fold change ≥ 1.5 and p < 0.05).

**Figure S19.** Therapeutic efficacy of once-daily BBR@MPN-NGs in the scopolamine-induced dry eye model. (A) Schematic of the scopolamine-induced DED model and treatment regimen. (B) Representative corneal fluorescein staining images over the 5-day treatment. (C) Corresponding fluorescein staining scores. (D) Tear volume measured by the phenol red thread test. (E) TBUT of the DED mice treated with different therapies. (F) PAS staining of conjunctival goblet cells and H&E staining of lacrimal glands at the end of treatment. Scale bar: 100 µm. Data are presented as mean ± SD (n = 6). Statistical significance was analyzed by the Kruskal-Wallis test (****p* < 0.001). Illustration in A was created using Adobe Illustrator.

**Figure S20.** Sterility testing and endotoxin detection. (A) Results of *S. aureus* (positive control) and BBR@MPN-NGs after 14 days of incubation in FTM and TSB, respectively. (B) Photographs showing gel formation in the LAL gel-clot assay for endotoxin detection in BBR@MPN-NGs, with LAL reagent water (LRW) serving as the negative control.

**Figure S21.** Cell viability of HCECs (A) and RAW264.7 cells (B) after incubation with different concentrations of BBR, MPN-NGs, and BBR@MPN-NGs, evaluated by MTT assay. Concentration of BBR@MPN-NGs: Calibrated by BBR and TA; MPN-NGs: Calibrated by TA.

**Figure S22.** Safety assessment of BBR@MPN-NGs. (A) Representative slit-lamp photographs of PBS- or BBR@MPN-NGs treated mouse eyes for up to 28 days using broad-beam lighting and fluorescein staining. (B) Representative H&E staining slices of the mouse cornea and lacrimal gland in different groups (scale bars: 50 µm). (C) Representative PAS staining images of conjunctiva in different groups (scale bars: 50 µm). (D) Quantitative analysis of the corneal thickness. (E) Quantitative analysis of the corneal epithelial thickness. Data are presented as mean ± SD (n = 3).

**Figure S23.** Zn accumulation in ocular surface tissues was quantified by ICP-MS at 0, 14, and 28 days following instillation of BBR@MPN-NGs in healthy mice (data from a single pooled sample of the cornea and conjunctiva from 6 mice per time point).

**Figure S24.** H&E staining of histological sections of heart, liver, spleen, lung, and kidney samples collected on the 28th day after diverse treatments, including 5 µL of PBS and BBR@MPN-NGs (BBR: 200 µM, TA: 681.2 µg mL^-1^) applied to the eye surface twice per day. The scale bar is 100 µm.

**References**

[1] X. Yuan, D. C. Marcano, C. S. Shin, et al., “Ocular Drug Delivery Nanowafer with Enhanced Therapeutic Efficacy,” *ACS Nano* 9 (2015): 1749-58. <https://doi.org/10.1021/nn506599f>

[2] R. Corrales, A. Villarreal, W. Farley, et al., “Strain-Related Cytokine Profiles on the Murine Ocular Surface in Response to Desiccating Stress,” *Cornea* 26 (2007): 579-584. <https://doi.org/10.1097/ICO.0b013e318033a729>
